# De novo design of anti-variant COVID-19 vaccine

**DOI:** 10.1093/biomethods/bpad021

**Published:** 2023-09-26

**Authors:** Arpita Goswami, Madan Kumar, Samee Ullah, Milind M Gore

**Affiliations:** Kshamalab, Leo’s Research Services and Suppliers, Mysuru 570016, India; Department of Chemistry-BMC Biochemistry, University of Uppsala, Uppsala 75237, Sweden; National Center for Bioinformatics (NCB), Islamabad 45320, Pakistan; 5/1B, Krutika Co-Op Housing Society, Kothrud, Pune 411039, India

**Keywords:** SARS-CoV-2, vaccine, spike protein, clinical T-cell epitopes, de novo protein design, folding driver epitope, HuCoV, AlphaFold, molecular dynamics simulations

## Abstract

Recent studies highlight the effectiveness of hybrid Severe Acute Respiratory Syndrome-Coronavirus-2 (SARS-CoV-2) vaccines combining wild-type nucleocapsid and Spike proteins. We have further enhanced this strategy by incorporating delta and omicron variants’ spike protein mutations. Both delta and omicron mark the shifts in viral transmissibility and severity in unvaccinated and vaccinated patients. So their mutations are highly crucial for future viral variants also. Omicron is particularly adept at immune evasion by mutating spike epitopes. The rapid adaptations of Omicron and sub-variants to spike-based vaccines and simultaneous transmissibility underline the urgency for new vaccines in the continuous battle against SARS-CoV-2. Therefore, we have added three persistent T-cell-stimulating nucleocapsid peptides similar to homologous sequences from seasonal Human Coronaviruses (HuCoV) and an envelope peptide that elicits a strong T-cell immune response. These peptides are clustered in the hybrid spike’s cytoplasmic region with non-immunogenic linkers, enabling systematic arrangement. AlphaFold (Artificial intelligence-based model building) analysis suggests omitting the transmembrane domain enhances these cytoplasmic epitopes’ folding efficiency which can ensure persistent immunity for CD4^+^ structural epitopes. Further molecular dynamics simulations validate the compact conformation of the modeled structures and a flexible C-terminus region. Overall, the structures show stability and less conformational fluctuation throughout the simulation. Also, the AlphaFold predicted structural epitopes maintained their folds during simulation to ensure the specificity of CD4^+^ T-cell response after vaccination. Our proposed approach may provide options for incorporating diverse anti-viral T-cell peptides, similar to HuCoV, into linker regions. This versatility can be promising to address outbreaks and challenges posed by various viruses for effective management in this era of innovative vaccines.

## Introduction

SARS-CoV-2 has brought the world to an unprecedented standstill for the last 4 years [1]. The massacre and chaos created are prevailing still. This infection demonstrates a wide clinical spectrum, from asymptomatic, mild, and moderate disease to even long-term positivity and mortality [2, 3]. Also, new variants are appearing regularly with mutations in anti-spike antibody binding sites causing new panic [4, 5]. Although, mortality in infected patients is lower than earlier SARS, serious further complications are seen in reinfected and even in vaccinated populations. These include myocarditis, damage to CNS, etc. that too in 30–40 years’ population [6]. Reinfection with emerging newer variants is also a cause of concern [7–9].

In this scenario question arises on the sustainability of vaccines based on only wild-type spikes [10]. The spike protein, which elicits neutralizing antibodies, is essential for recovery [11]. However, the problem is the short term of the direct mucosal immune response. Long-term memory in mucosal immune response is generated by T-cell response [12, 13]. In children older than 2 years, infections by closely related coronaviruses (common cold) generate mild and asymptotic responses against OC43, HKU1, 229E, and NL63 (HuCoV) [14, 15]. Several immune response studies on these HuCoV infections have been carried out [16–18] so far. By the age of 2–5 years, 80% of the children are immunized and have persistent neutralizing antibodies for life [16]. The associated T-cell response is also highly effective in generating memory response and lower severity in infected COVID patients [19–22]. Several studies also suggest that T-cell immune response inducing nucleocapsid (N) protein has to be included in potential corona vaccine strategy pipelines [23–26]. This can be a promising approach to curb circulating Delta [27], Omicron variants [28], and other recombinants [29]. Both Delta and Omicron (BA.1) are crucial as they mark the transition between pre-vaccination and post vaccination immune responses. Therefore, it is important to design a vaccine with delta–omicron spike protein and T-cell epitopes. For this, we have designed a construct using chimeric SARS-CoV-2 spike and SARS-CoV-2 T-cell epitope sequences. Structural proteins with almost conserved sequences for different coronavirus families were targeted for potential T-cell enticing candidates. Functional proteins were not considered since some of them are known to hijack MHC systems in humans [30–32]. Several studies previously showed the homology of nucleocapsid protein sequences between SARS-CoV-2 and HuCoV strains [33–35]. Also, the T-cell response of SARS-CoV-2-unexposed individuals to SARS-CoV-2 was previously reported for one peptide from the envelope (E) protein [36] indicating potential effect from common cold immunity. Therefore, we exclusively focused on nucleocapsid protein and envelope protein for T-cell epitopes and compared them with convalescent patients’ T-cell response-inducing sequences from the literature. More attention was given to CD4+ TCR epitopes than CD8^+^. This is because the former sequences were found to be more stable than the latter, according to studies on patients [37]. The designed sequence had four epitopes joined by linkers as cytoplasmic regions. Among these, the two epitopes were found folded when modeled without transmembrane domain. More well-formed structural epitopes can potentially ensure greater CD4^+^ T-cell activity [38], leading to sustained immunity. Lastly, the conformational stability and dynamics of the designed structures are confirmed using the molecular dynamics (MD) simulation (MDS) at 100 ns time. Thus, we propose that this sequence and the structures provided by our strategy could be quite useful and should be considered for vaccine design and development against SARS-CoV-2 and can also be easily tweaked to any other future variants, respiratory and other viruses with the potential to cause pandemic.

## Materials and methods

**Figure 1.**
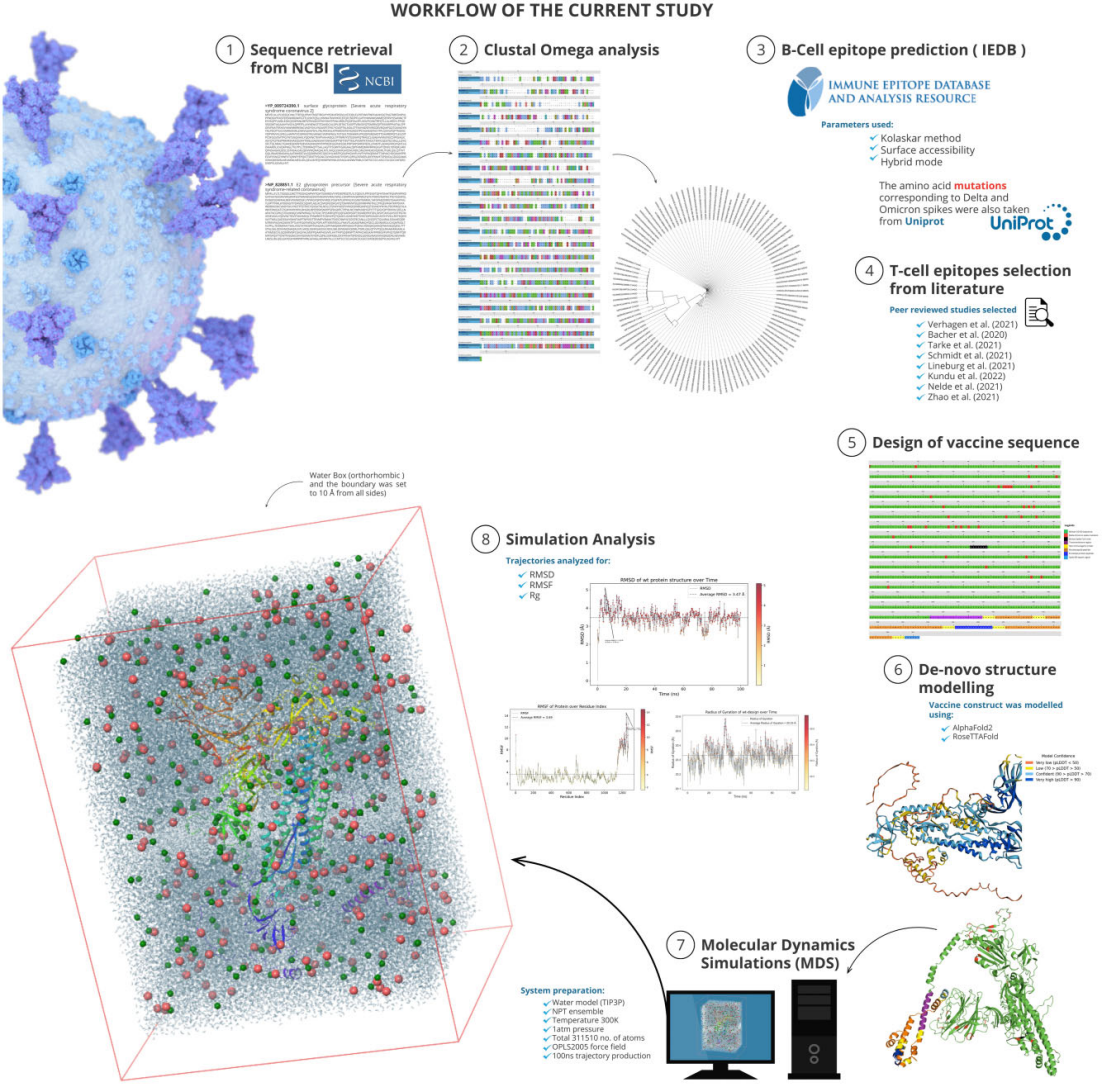
Graphical Abstract of the study.

The complete workflow of the current study is provided in Fig. 1.

### Sequences retrieval

The SARS-1 and SARS-CoV-2 spike sequences were retrieved from the UniProt database (Universal Protein Resource) with accession no. (P59594 and P0DTC2) [39]. Sequences for SARS-CoV-2 and other structural proteins, such as nucleocapsid (P0DTC9), envelope (P0DTC4), and membrane (P0DTC5), including the homologous structural protein sequences for HuCoV and SARS-1 were also retrieved from the UniProt database (RRID: SCR_002380).

### Clustal omega analysis

These sequences were imported into the Clustal Omega server of the European Bioinformatics Institute (EBI; RRID: SCR_001591) for sequence alignment using the default protocol of ClustalW with characters count. Before running the alignment, the values for parameters of guide tree iterations, Hidden Markov Model iterations and combined iterations were set to the maximum (default value −1) [40].

### B-cell epitopes prediction

The SARS-1 and SARS-CoV-2 spike sequences were then imported into the (IEDB-Immune Epitope Database) server (RRID: SCR_006604), and the B-cell epitopes predicted [41]. The prediction parameters were set to the Kolaskar method, surface accessibility, and a hybrid mode. The predicted B-cell epitopes were color-coded based on the method shown (Fig. 4). The individual protein domains were also mapped on the aligned sequences using information from both Clustal alignment and UniProt. The amino acid mutations corresponding to Delta and Omicron spikes were also retrieved from the UniProt. All these mutations are incorporated in the native SARS-CoV-2 spike sequence and a hybrid spike, a single sequence containing both the Delta and Omicron mutations achieved. The native furin site (PRRAR) is kept due to its significance as a hotspot for immune response.

### T-cell epitopes selection from literature

The T-cell epitopes from SARS-CoV-2 recovered convalescent patients’ peer-reviewed studies were selected from available extensive literatures [17, 18, 20, 31, 32, 34, 40, 41]. Because the CD8^+^ epitopes cause non-persistent immunity, in this study, the literature was searched for CD4^+^ epitopes [37] and the potent epitopes with the best possible folds of secondary structures as per PDBsum selected. Nucleocapsid and envelope peptides were preferred, including one memory peptide (with both T-cell and B-cell immunity). The similarities with homologous proteins from the common cold coronavirus families as per literature studies were also included. The peptides having similarity to human proteins including the closely related regions in individual open-reading frames were excluded from this study to avoid allergenicity [42]. Finally, to prevent misfolding in the design, the secondary structures of the selected peptides were predicted using the PDBsum (RRID: SCR_006511) and Phyre2 (RRID: SCR_010270) [43, 44].

### Sequence conservation analysis

Clustal omega sequence alignments for these proteins were generated using the UniProt data on SARS-CoV-2, SARS, and common cold coronavirus. The alignment was refined by removing gaped sequences, non-human host harboring coronavirus homologs and partial protein fragments. The final alignment and associated sequence similarity based on neighbor joining tree (without distance corrections) were generated with a maximum of five iterations. Finally, the guide tree was imported and plotted in the iTOL phylogenetic tree visualization server (interactive Tree of Life, ver. 6.5.8, EMBL) (RRID: SCR_018174) [45].

### Design of vaccine sequence

The hybrid sequence had mutated amino acids from both Delta and Omicron (BA.1) spikes. The core β-strand was deleted from the S2 region to prevent pre- to postfusion transition as shown previously for coronavirus spike transitions (Fig. 6) [46]. Potential antibody-dependent cellular cytotoxicity/antibody-dependent enhancement (ADCC/ADE) segment [47–50] was mapped from Clustal Omega (Fig. 4) and further fine-tuned by structural alignment with *SARS-1* spike in PyMOL (Fig. 5) (RRID: SCR_000305). The smallest region that maintained stability was removed after confirming in the AlphaFold-disorder package [51]. The N-epitopes interconnected by non-immunogenic EAAAK linkers were incorporated into the cytoplasmic domain [52–55]. These epitopes were incorporated based on their native position in the nucleocapsid structure. The envelope peptide (15 residues) is placed between the linkers among the last two N-epitopes to aid in the interaction (to cancel zero neighbors’ interactions effect from rigid EAAAK linker helices). The interaction of these two N-epitopes with each other in the structure can be seen in Supplementary Fig. S6.

### De-novo vaccine structure modeling

The resulting sequence was imported into the Robetta server which uses the RoseTTAFold algorithm to build protein structures and using the default settings the vaccine structures were modeled (RRID: SCR_015701). In addition, the model building was also achieved using the AlphaFold (version 2.0) in the Uppsala server (UPPMAX, Uppsala Multidisciplinary Center for Advanced Computational Science, Uppsala University, Sweden) (RRID: SCR_023662) [56]. The PyMOL (PyMOL-Molecular Graphics System, Open-Source version, Schrödinger, LLC) and the alpha-viewer python package (Severin Dicks, IBSM Freiburg) were used for the visualization and analyses of the structures. The models for the hybrid spike without transmembrane domain and the wild-type of SARS-CoV-2 spike were also generated by AlphaFold. Furthermore, all three models were compared using the predicated template modeling mode to obtain predicated aligned error (PAE) map. Finally, the predicted local distance difference test (pLDDT) plots were also calculated for all models and carefully checked the pLDDT values which show per residue confidence scores for the accuracy of the models.

## MDSs protocol

### System preparation and trajectory production

The 3D structures of the final models in this study (1273, 1291, and 1312 amino acid residues) were prepared using the default protocol of PlayMolecule ProteinPrepare [57]. The protonation states of all model residues at pH 7.0 were determined by PROPKA v3.1 [58]. The missing atoms were added, the bond orders corrected, and the H-networks (atoms) were optimized by PDB2PQR v2.1 (RRID: SCR_024155) [59]. The structures were minimized using the YASARA minimization server and the resulting structures were used for the MDS’s analysis (RRID: SCR_017591) [60].

The MDS of the systems was performed for 100 ns in Desmond (RRID: SCR_014575) using the TIP3P (transferable intermolecular potential 3P) water model as the solvent [61]. The protein structures were placed in an orthorhombic box with a minimum distance of 10 Å between the proteins and the box edges and solvated with water molecules and 0.1 M (molar) NaCl. The systems were neutralized by adding 08 Na(sodium) ions for wild-type and 05 Na ions to other structures (Vac^FL^ and Vac^deltm^). The temperature and pressure were monitored using the NPT ensemble of Berendsen thermostat 300 K and barostat 1 atm for the structures [62]. The production run was set to 100 ns time, and the trajectories were recorded using the OPLS2005 force field [63]. The total number of atoms in the full systems was checked and noted as 311510 (wild ^type^), 407960 (Vac^deltm^), and 329369(Vac^FL^) atoms, respectively.

### Simulation analysis

The trajectories were analyzed for various structural and dynamic properties, including the root-mean-square deviation (RMSD), root-mean-square fluctuation (RMSF), radius of gyration (Rg), and the time-based gradients analysis of the structures.

## Results

### UniProt analyses

**Figure 2.**
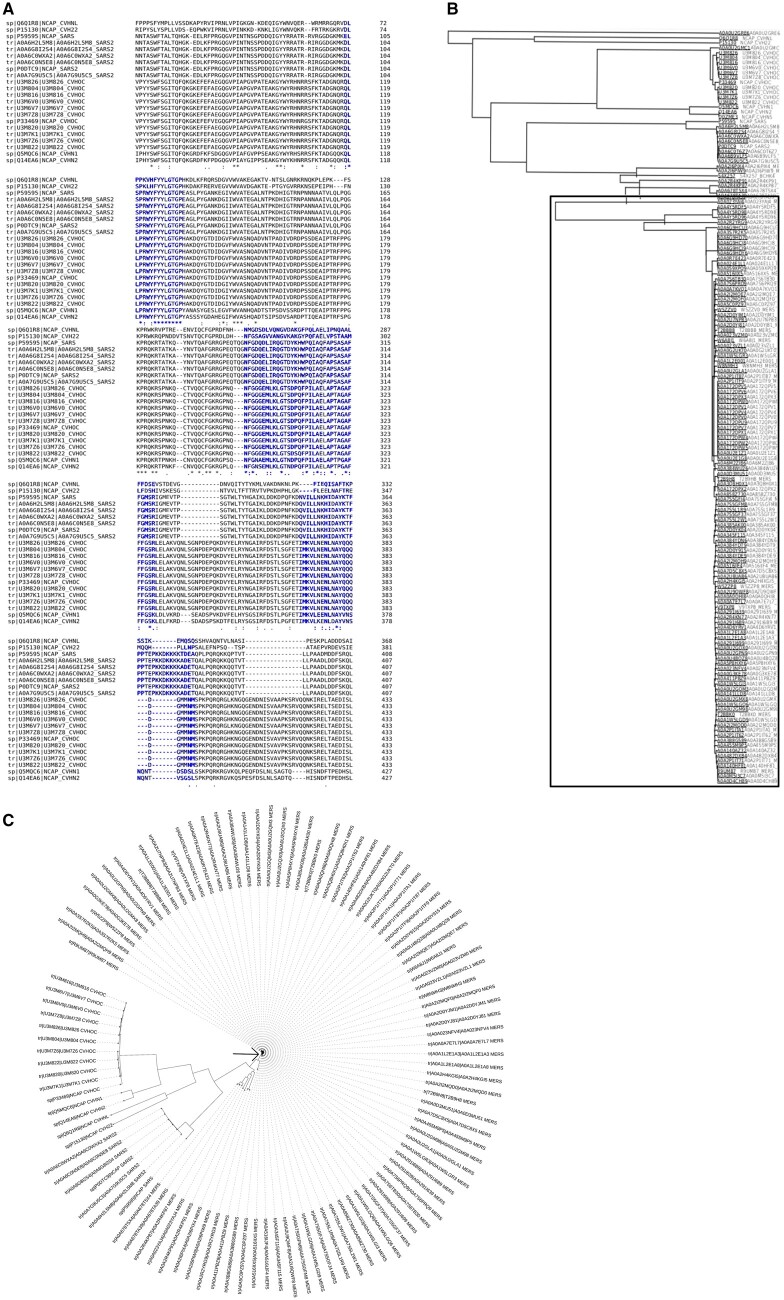
Clustal Omega alignment and sequence similarity-based guide tree of Coronavirus nucleocapsid (N) sequences. (A) The Clustal Omega sequence alignment among SARS, COVID, and common cold coronaviruses shows high similarity in chosen epitopes (blue). Except for the last several residues in the last peptide, all SARS2 N-epitopes are highly similar to HuCoV sequences. (B) Preliminary guide tree from Clustal Omega alignment of nucleocapsid sequences is shown. UniProt-retrieved Coronavirus N-protein sequences’ alignment shows distinct unique tree formation from later variants of MERS (box enclosed). Here all sequences available from the database are used. (C) Sequence similarity-based neighbor-joining circular tree of coronavirus nucleocapsid proteins sequences. These are chosen from UniProt after removing fragments, gaped sequences, and nonhuman hosts infecting coronaviruses. For SARS, only one sequence was found to be without gaps. The circular tree is plotted in iTOL. Arrow marked is MERS nucleocapsid proteins showing slight changes in their sequences when compared to others.

UniProt mining of SARS-COV-2 structural proteins (spike, nucleocapsid, membrane, and envelope) was divided into three parts: (i) Retrieval of SARS-CoV-2, SARS, common cold and MERS sequences, (ii) Clustal Omega alignment of each structural protein from UniProt, and (iii) Sequence similarity-based neighbor-joining tree generation (without distance values) in Clustal Omega. At first, sequences corresponding to coronaviruses infecting all host animals were considered. For nucleocapsid sequences, we found that the addition of MERS nucleocapsid generates a distinct tree which slowly departs from another corona including SARS-CoV-2 (Fig. 2B). Furthermore, we generated a circular tree by filtering out gaped sequences, protein fragments, and non-human host infecting coronaviruses. This confirmed that MERS nucleocapsid protein sequences indeed form a distinct tree, but this is due to them being more conserved compared to others (Fig. 2C). Usually, the nucleocapsid protein is more highly conserved than the spike protein [23] and is more immunogenic [35]. It is also essential for packaging the RNA structure inside the viral particles. If random mutations arise in the nucleocapsid, the virus will possibly not survive in the long run. Considering this scenario with MERS, the introduction of recurrent random mutations in the nucleocapsid protein, potentially driven by immune responses, could be beneficial for vaccine design. Although the SARS-CoV-2 (SARS-2) nucleocapsid has more mutations than MERS, the frequency is still much lower compared to the spike protein (Fig. 2C;Supplementary Fig. S1). Alongside that we also generated similar sequence-similarity trees with other structural proteins (Spike, Membrane, and Envelope) (Supplementary Figs. S1–S3). The focus was on two conditions: (i) Conserved structural proteins among extant coronaviruses (MERS, COVID, and common cold coronaviruses; not SARS, as it is no longer circulating among humans); (ii) How immunogenic these proteins are. We found out that envelope protein can also be a suitable candidate like nucleocapsid. These protein sequences were highly conserved among SARS-CoV-2 strains (Supplementary Fig. S3). Thus, we selected a non-allergenic epitope from the envelope protein. This epitope is crucial for binding to human cell junction proteins [64] and it elicits a robust immune response [36]. Surprisingly, spike protein sequences were also found highly conserved among MERS (Supplementary Fig. S1). As this has already been selected for use in existing vaccine candidates, including our own design, we did not further investigate this particular aspect. Although the membrane protein (M) was found to be highly conserved among extinct SARS strains (Supplementary Fig. S2), it was not chosen due to its lower immunological persistence (described below).

### Literature hunting for potential T-cell epitopes for vaccine design

Immunogenic sequences of COVID nucleocapsid and envelope proteins were carefully shortlisted from literature studies. First, the sequences with overlapping CD4+ and CD8+ epitopes were preferred. Similarities with common cold viral sequences were also considered (80) (Fig. 2A). The data available from recovered and asymptomatic populations were given the highest importance. The final selected peptides were screened based on their structures (from PDBsum) and secondary structure predicted by the Phyre2 server (in the absence of experimentally solved structure for envelope protein; Supplementary Fig. S7). Because SARS-2 CD8^+^ epitopes are hotspots of nonsynonymous mutations to cause short-lived immunity and mostly unstructured, therefore the epitopes with definite structures were preferred to invoke CD4-based immunity. Subsequently, two epitopes were found to be structurally folded in the AlphaFold models. However, we found one epitope fully and another partially non-structural after validation in Alpha Fold as well. These were kept in the final construct based on their ability to provoke rapid immune response due to high similarity with common cold homologs and strong memory response. The membrane protein epitopes were found to be less persistent as per the literature study apart from highly COVID-exposed hospital workers who are more immune to COVID compared to other populations. The selected peptides are described in the Supplementary Table S1.

### RoseTTAFold building and structure validation in AlphaFold

The designed sequence had a signal peptide, transmembrane domain, and a cytoplasmic domain formed by T-cell epitopes interconnected by EAAAK linkers. A major stabilizing portion of putative antibody-dependent cell-mediated cytotoxicity/antibody-dependent enhancement (ADCC/ADE) region and core β-strand were removed. The resulting local disorder in the remaining ADE segment after deletion was confirmed from analysis in the AlphaFold-disorder package (Supplementary Fig. S11) which can predict residue fluctuations from pLDDT values and relative solvent accessibility. The positively charged endoplasmic reticulum (ER) exit signal from the COVID native spike was placed at the cytoplasmic end to help easy exit from the ER. This sequence was first modeled in RoseTTAFold and found to be structurally similar to the native spike. All domains, including the cytoplasmic domain, are distinctly well-formed (Fig. 7). To confirm further, we have built AlphaFold models of the two sequences: with transmembrane region (TM) (Vac^FL^) and without TM (Vac^deltm^). It was observed that at least two epitopes (NFGDQELIRQGTDYKHWPQIAQFAPSASAFFGMSR and FYVYSRVKNLNSSRV) were folded in the Vac^deltm^ sequence (Fig. 8). The first sequence is well-folded in the Vac^FL^ (with transmembrane) construct, unlike the other (Fig. 10). It appears the first sequence is the major driver of folding for the designed cytoplasmic domain. Additionally, inter-residual distance analysis in PyMOL showed a longer distance (21 Å) between this peptide and the final sequence, also referred to as the memory epitope (QVILLNKHIDAYKTFPPTEPKKDKKKKADET) (81), in the Vac^FL^ construct compared to the corresponding distance (8.5 Å) in the other construct (Fig. 9). The other peptide (DLSPRWYFYYLGTGP) and memory epitope are fully unstructured in Vac^FL^ construct. The memory epitope (specific region of an antigen recognized by antigen-specific memory lymphocytes) is partially structured in Vac^deltm^ possibly due to the closer distance (8.5 Å) (Fig. 11A) to the folding driver peptide. Apparently, this closeness resulted from an absence of transmembrane domain in Vac^deltm^ which otherwise pulled away (Fig. 9B) the folding driver epitope (FDE) from the memory epitope in Vac^FL^ model. This might have caused unstructured memory peptide in Vac^FL^ construct. In fact, both Vac^FL^ model and native spike (having transmembrane region) have shown mostly unstructured cytoplasmic domain which is further confirmed by the PAE scores similarities (Supplementary Figs. S9 and S10) in this domain for these two models. For the main protein, the receptor-binding domain (RBD) is found to be well-folded and identical among all three models including the native spike (Supplementary Fig. S4). Also, the core β-strand is replaced by two discontinuous stretches of β-strands which can form hydrogen bonds with the complementary strand in the core β-sheet (Supplementary Fig. S5). Thus, the structural integrity of this region can be maintained, while the lengths of these strands are not enough to support pre-to post fusion transition of spike, unlike native core β-strand.

### Design validation through MDS

One of the challenges of de novo design of anti-variant COVID-19 vaccines is to ensure that they elicit robust and long-lasting T-cell memory response. This can be achieved if the designs are structurally stable. Therefore, in this study, we further confirmed the AlphaFold designs in 100 ns of MDSs based on various parameters of RMSD, RMSF and Rg, and assessed the structural stability and dynamics of the designed vaccine scaffolds. These parameters can provide insights into the conformational changes, flexibility, and compactness of the structures over time for indicating stable design. At first, simulation predictions from wild-type COVID spike were analyzed as a reference. Figure 11A shows the RMSD plot of the wild-type spike protein backbone atoms as a function of time. The RMSD is a measure of how much the protein structure deviates from a reference structure, usually the initial or native structure. The initial RMSD value at 5.30 ns recorded 5.09 Å, indicating a large deviation from the starting structure. This could be due to the initial equilibration of the system and the relaxation of the protein conformation during simulations. Later, the RMSD values decreased and fluctuated around an average value of 3.47 Å, suggesting that the protein reached a stable conformation and did not undergo significant structural changes during the rest of the simulation. The protein structure is determined by a balance of different forces, including covalent bonds, Van der Waals interactions, hydrogen bonds, salt bridges, and electrostatic interactions. Electrostatic interactions are caused by the presence of charged or polar residues on the protein surface or in the interior. The electrostatic interactions can affect protein structures in several ways. They can either stabilize or destabilize the protein fold by forming favorable or unfavorable interactions with other residues or with the solvent molecules. They can also modulate the flexibility and dynamics of the protein by influencing the rigidity or mobility of certain regions or domains. The histogram in Fig. 11B shows the electrostatic interaction values between 0 to −0.3 kcal/mol for the wild-type spike protein structure at 100 ns time. The electrostatic interaction value is calculated as the sum of Coulombic interactions between all pairs of charged or polar atoms in the protein, divided by the number of atoms. A more negative value indicates overall attractive, while a positive value indicates an overall repulsive. The histogram shows that most of the electrostatic interaction values are close to zero, indicating that there is a balance between attractive and repulsive interactions in the protein structure. However, there are some outliers with more negative values, indicating that these regions or residues have somewhat stronger electrostatic interactions than others. These residues with more negative values may be involved in stabilizing salt bridges or H-bonds with other residues and regions and they could be more rigid or flexible than others, affecting their conformational changes or dynamics at 100 ns of time trajectory. The RMSF analysis is a method to measure the flexibility of different regions of a molecular structure over time. It can reveal the more stable or highly dynamic residues or regions of the structure contributing during MDSs. Figure 12A shows the RMSF value of each residue along the wild-type spike protein sequence. The residues with higher RMSF values are colored in red, indicating that they are more flexible than the residues with lower RMSF values, which are colored in gray. The highly flexible single residue is shown at the 1246 position with an RMSF value of 14.48 Å and is found at the C-terminus of the protein, which is expected to be more mobile than the rest of the core structural residues. In addition, the C-terminal region, starting from 1160-THR to the end, has an overall high RMSF value, suggesting that it is a very flexible segment shown by red color representation in Fig. 12A. Also, the average RMSF value of 3.69 Å for overall structure in 100 ns time of simulations is consistent with the histogram in Fig. 12B, which shows the distribution of RMSF values indicating the significance of the C-terminal region in terms of flexibility and conformational transitions. The Rg is a measure of how the atoms of a protein are distributed around its center of mass. The smaller the Rg, the more compact the protein structure is. In Fig. 13A–B, the Rg of the structure was calculated for each frame of 100 ns MDS. The highest value was 20.59 Å and the average value was 20.33 Å, indicating that the wild-type spike protein maintained a relatively compact conformation throughout the simulation. To visualize the structural changes over time, we plotted the time-based gradients of these structures in Fig. 14, where the blue color represents the initial structure, and the red color for the final structure. The structure underwent shrinkage during the simulation, which could be due to the formation or stabilization of intra or intermolecular interactions. The 3D representation of the protein structures at different time points during the simulation can be seen in Fig. 14. The structures are aligned by their centers of mass and colored according to their time and order. The blue structure corresponds to the initial conformation at 0 ns, while the red structure corresponds to the final conformation at 100 ns frame. The intermediate structures are colored with a gradient from blue to red, indicating their relative position in time.

The RMSD Analysis of the trajectories in Fig. 15A–D for both vaccine constructs (Vac^deltm^ and Vac^FL^) revealed that the scaffolds maintained stable folded conformation (average RMSD values of 1–1.3 Å with the highest 1.98 Å and 1.34 Å at a time trajectory of 14.9 ns and 52 ns, respectively) indicating limited deviations from the starting structures. These distributions can also be observed in the histogram representations corresponding to the trajectories. The dynamic flexibility in terms of RMSFs in both vaccine scaffolds also indicated stable folded conformation in Fig. 16A–D, while Vac^FL^ showed a slightly higher average 2.9 Å of fluctuations compared to Vac^deltm^ (2.38 Å). Furthermore, in Vac^deltm^, the N-terminus at position 1 (MET) is found to be more flexible (8.54 Å) when compared to Vac^FL^. While in the latter, the C-terminus at position 1257 residue showed higher fluctuations (10.19 Å) than the former. But overall, in both cases, the C-terminus indicated higher flexibility and was prone to structural changes throughout the simulations. This further indicates that the C-terminus in both vaccine scaffolds is more accessible to solvents compared to internal core regions throughout the simulations signifying this region may confer greater plasticity to enable immune interactions and epitope presentation. In Fig. 17A–D, the global sizes and the compactness in terms of Rg in both vaccine scaffolds remained stable (Rg distribution centered around 48–49 Å), confirming their properly folded shape. Hence the designed vaccine structures firmly maintained their overall geometry throughout the simulations (Supplementary movies). For the cytoplasmic domain, FDE-mediated folding is maintained in Vac^deltm^ (Fig. 15A and B; Supplementary Fig. S12) after 100 ns. In Vac^FL^, slightly more folding was observed for the memory peptide (Fig. 15C and D; Supplementary Fig. S12) but no folding was achieved for the envelope peptide. Therefore, the 100 ns simulations of the Vac^deltm^ and Vac^FL^*de novo*-designed vaccine scaffolds provided critical validation of the AlphaFold structures’ folding stability and favorable dynamics. Both designs robustly maintained their folded conformations with only modest fluctuations localized to the terminal regions evidenced by its lower overall RMSD, RMSF and Rg deviations. The observed structural flexibility balanced between the stable cores and solvent-exposed terminals aids in conformational plasticity while resisting misfolding. Overall, the favorable stability and flexibility properties demonstrated by the rationally designed Vac^deltm^ and Vac^FL^ constructs provide a promising framework for structure-based vaccine engineering against challenging pathogens and diseases.

## Discussion

**Figure 3.**
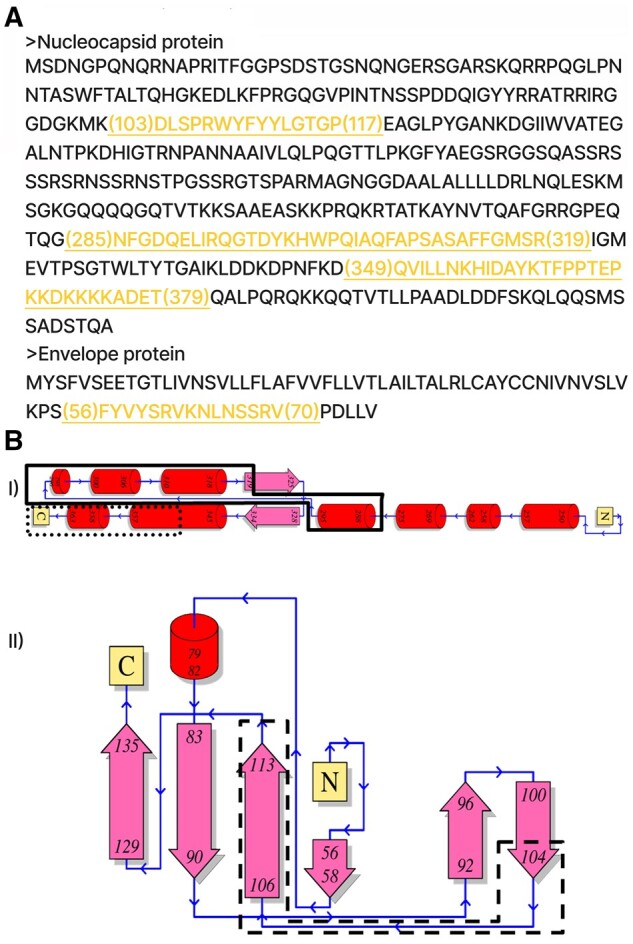
Wuhan COVID nucleocapsid and Envelope protein epitopes. (A) The T-cell epitopes from N and E protein sequences (in FASTA format), homologous with common cold were selected for the cytoplasmic domain design of the vaccine construct. These are highlighted in yellow and underlined. The numbers in parentheses denote amino acid positions. (B) Secondary structures of the selected nucleocapsid peptides from PDBsum: (I) QVILLNKHIDAYKTFPPTEPKKDKKKKADET (PDB ID 6ZCO) in dotted rectangle has mostly α-helical structure with adjoining loops; 15 residues missing at C-terminus. NFGDQELIRQGTDYKHWPQIAQFAPSASAFFGMSR (PDB ID 6ZCO) in continuous lined enclosure has α-helical structures with adjoining loops. (II) DLSPRWYFYYLGTGP (PDB ID 7N0R) in the dashed enclosure has non-complementary β-strands along with adjoining long loop thereby strong possibility of forming unstructured epitope in construct design. The core sequence in this epitope is SPRWYFYYL which induces CD8+ response (Supplementary Table S1). CD8+ epitopes are usually linear/unstructured.

**Figure 4.**
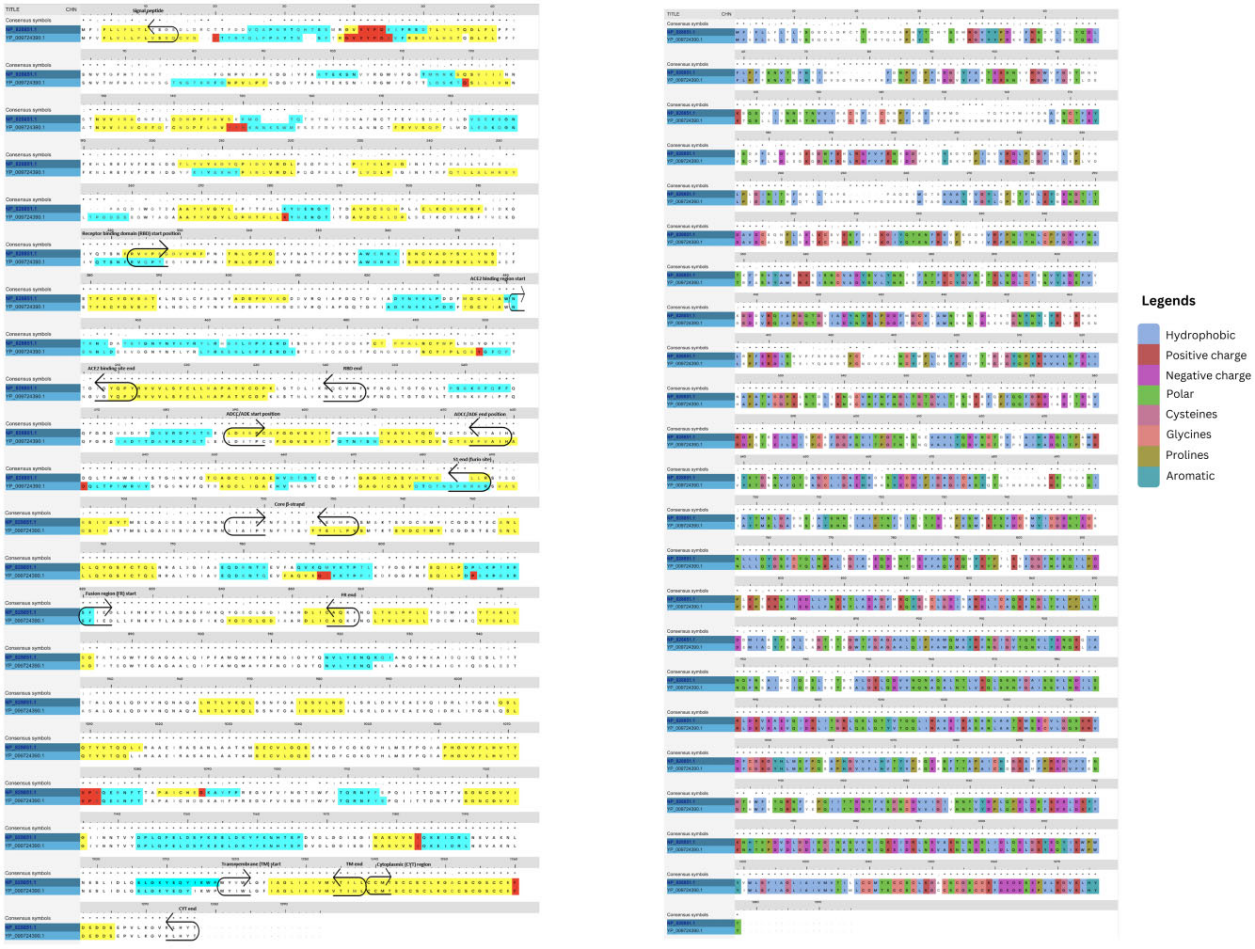
Mapping of B-cell epitope regions of Wuhan COVID spike (accession no. YP_009724390.1). Native SARS2 spike RBD, Angiotensin-converting enzyme 2 binding sequence, furin site, core β-strand, and fusion region (FR) along with ADCC/ADE inducing regions are aligned with SARS spike (accession no. NP_828851.1) in Clustal Omega. The B-cell epitope analysis is from the IEDB server. Yellow is B-cell epitopes by the Kolaskar method, cyan blue is surface accessibility. Red has both methods. These B-cell epitope regions are present in delta and omicron spikes also with frequently changing sequences evading immunity. Also mapped on the right side with the color scales based on amino acid properties.

**Figure 5.**
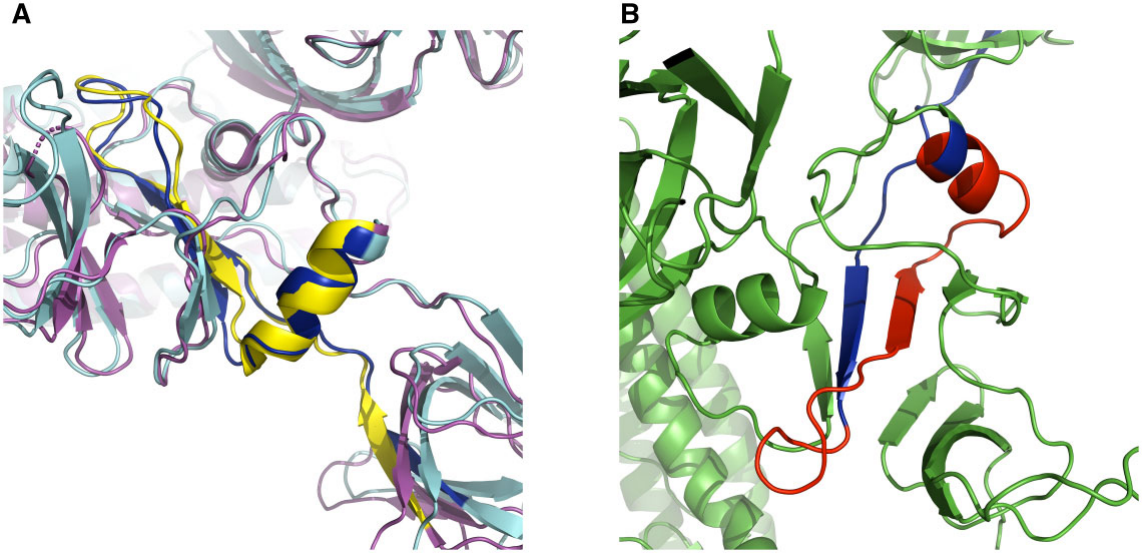
Mapping of ADCC and ADE region on COVID spike in PyMOL. (A) Clustal Omega mapped region (585–625; blue) for COVID spike (cyan) is investigated by structural alignment on ADE inducing region (yellow) in SARS Spike (magenta). (B) In vaccine construct design, red segment (Δ600–624) was deleted to sufficiently weaken that region (rather than a beta cage to encase any possible immunological proteins) as predicted by AlphaFold-disorder analysis. Other non-ADE sequences are colored green.

**Figure 6.**
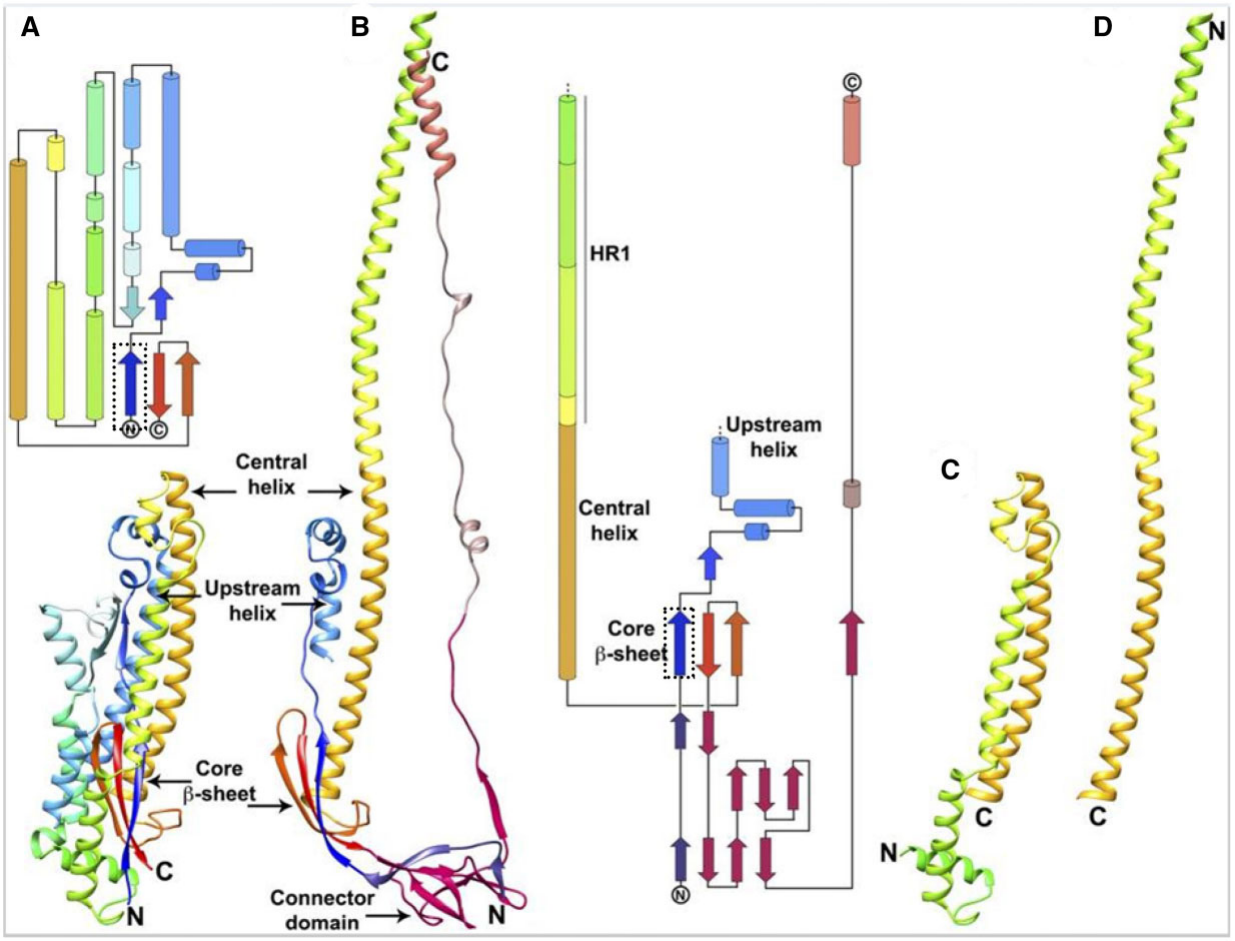
Core β-strand (dark blue, in dotted rectangle, residues: 711–729) is essential for pre- to post-fusion transition of spike. This strand maintains the core β-sheet by exchanging interactions with the other two strands from same protomer to homologous strands of different protomers of trimeric spike. (A) Pre-fusion spike S2 apparatus comprising central helix, HR region, and core β-sheet. (B) Post-fusion spike S2 apparatus comprising central helix fused with HR region; Core β-sheet with exchanged second and third strands between protomers. Core β-strand remains unchanged. (C) and (D) Central helix and HR1 in pre- and post-fusion conformation. [Reprinted with permission from PNAS] [46].

In this study, we computationally designed two vaccine models (Vacdeltm and VacFL) with the potential to overcome SARS-CoV-2, a highly infectious virus. We further confirmed their conformational stability and fluctuations using the MDS. The conserved structural proteins along with the spike were targeted for the design of potential vaccine constructs. The designed structures were validated based on various computational parameters in 100 ns simulations. Our vaccine constructs are designed based on SARS-CoV-2 structural proteins’ T-cell response-inducing epitopes, as identified in the literature (Supplementary Table S1). The hallmark of current COVID-19 vaccination is wild-type spike protein because of its potent antigenicity. But the presence of vaccine-evading variants due to spike mutations led us to incorporate the mutant spike in our design. For example, the deadly Delta variant from the pre-vaccination era was followed by milder Omicron variants after mass vaccination. This massive paradigm shift in viral strains predicted future variants will be more likely recombinants to evade vaccine-elicited immunity. Therefore, hybrid spike sequence with both Delta and Omicron mutations is important for the potential design of vaccines to tackle SARS-CoV-2 variants which we achieved in our design. Particularly, our proposed strategy of vaccine scaffold integrates the mutated spike proteins, like other recently proposed variant-specific vaccines [65–67]. However, rather than just updating the spike antigen with hybrid Delta–Omicron mutations, we also integrate conserved CD4^+^ T-cell epitopes from nucleocapsid and envelope proteins based on their conservation across coronavirus strains [36, 68, 69]. In literature, dual-antigen vaccines using spike and nucleocapsid proteins have shown promise in eliciting broader immunogenicity [70, 71]. Our design further refines this approach by incorporating specific immunodominant epitopes in the spike cytoplasmic domain. This modular insertion of T-cell peptides with structural support from FDE is a unique aspect of our vaccine. It provides a versatile framework to induce robust CD4^+^ memory T cells against conserved epitopes from multiple SARS-CoV-2 proteins [72–74]. Furthermore, spike protein’s S2 segment has regions that do not generate neutralizing antibodies and do not have the dominant T-cell response. There is also a short, unstructured cytoplasmic domain that induces non-specific immune response in patients [75]. Our designs replaced it by inserting COVID’s T-cell epitopes interconnected by non-immunogenic, helical and rigid linker sequence EAAAK. To incorporate all these linkers, we have also removed segments of core β-strand (buried deep inside spike core, structurally dispensable in pre-fusion spike and immunologically less significant) (Figs 4 and 6) from the S2 region of spike. This could be significantly beneficial in vaccine design, allowing for the addition of new segments to the full molecule without significantly increasing its size. This also might help in reducing synthesis costs and time, while still preserving the original conformation of the molecule. Additionally, the absence of this region can prevent the spike from going from pre- to post fusion conformation (Fig. 6). Therefore, we did not consider the standard practice of proline–proline (a non-viral sequence) insertion, which is used in other spike-based vaccines for stabilization of pre-fusion spike [76]. Since the effect of this insertion in our core β-strand truncated constructs cannot be speculated without experimental validation. Also, we removed the putative region in S1 known to cause ADE responses in SARS, according to the literature and structural alignment (Fig. 5B) [47–50]. To select T-cell epitopes for the construction of the cytoplasmic domain, non-allergenic peptides were chosen from among the many T-cell epitopes present in the nucleocapsid protein (Fig. 3A). These selected peptides included a memory epitope that can elicit strong immune responses in patients who have recovered from or been vaccinated against the virus. These sequences are also highly similar to other common cold coronavirus (HuCoV) nucleocapsid protein sequences (Fig. 2A). All humans have an immune response against HuCoV from childhood driven by pulmonary immune memory including CD4^+^ epitopes. Therefore, using these epitopes has the advantage to generate long-term and quick immune responses. This establishes their importance in persistent immunity. Whether the selected peptides cause misfolding in the generated construct or not, the PDBsum analysis helped to screen nucleocapsid peptides that were found to fold with correct secondary structures (Fig. 3B). For envelope peptides that lacked experimentally solved protein data bank structure, we used Phyre2 structural predictions (Supplementary Fig. S7). Subsequently, model building in Rosetta Fold showed these peptides adopting alpha-helical fold in the designed cytoplasmic domain. Final AlphaFold model building predicted that at least two peptides are folded while the other two epitopes are mostly unstructured in construct without transmembrane region. One of the folded epitopes can be the main player in folding for other subsequent peptides. The predicted LDDT values were >70 for this epitope in both Vac^deltm^ and Vac^FL^ sequences (Figs 8 and 10). Also, we found out that the transmembrane domain pulls the adjacent region including the above-mentioned principal epitope for folding in the cytoplasmic domain from the other portions in Vac^FL^ construct. The distance between the last epitope and the FDE in the Vac^FL^ construct is significantly greater than in the Vac^deltm^ construct. Interestingly, the folding driver peptide was constructed by joining numerous CD4^+^ and CD8^+^-responsive N-peptides from immunological studies (Supplementary Table S1). In native nucleocapsid proteins, they are located as flanked to each other, parsed by the immune system as numerous small peptides. Apart from immunological significance, they might play a role in the folding of native nucleocapsid around viral RNA (ribonucleic acid). Parallel MD in 100 ns simulation time revealed stable conformations of the designed structures in terms of RMSD, initially higher and then onwards much more stable and very flexible C-terminal region in the RMSF. Furthermore, Rg and time-based-gradient analysis revealed a compact system suggesting overall conformational stability of the designed structures and less mobility or fluctuation transitions except for highly flexible C-terminal regions which otherwise will be fragmented by the immune system once recognized as potential epitopes.

**Figure 7.**
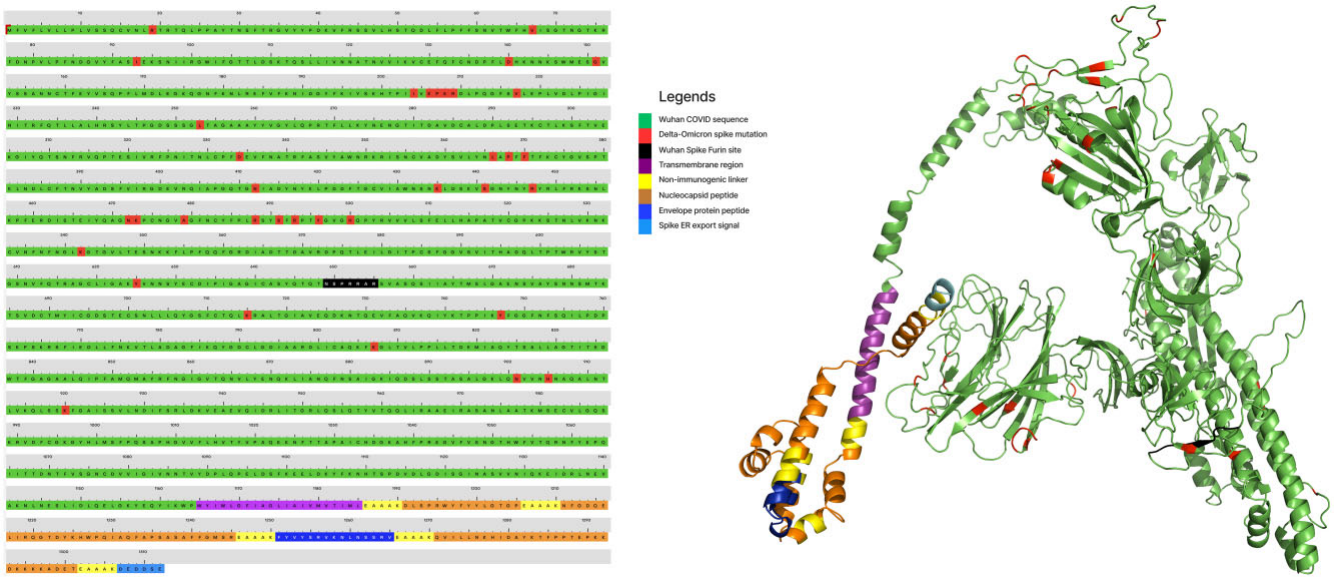
Sequence and 3D model (RoseTTAFold) of the VacFL vaccine construct. The mutations corresponding to Delta and Omicron variants are colored red. Transmembrane domain, linkers, cytoplasmic T-epitopes, and terminal ER exit signals are colored differently. All color codes are explained in the legend on the right side. The T-epitopes appear helical in this model while the cytoplasmic domain adopts a well-folded coiled coil located close to the main protein.

**Figure 8.**
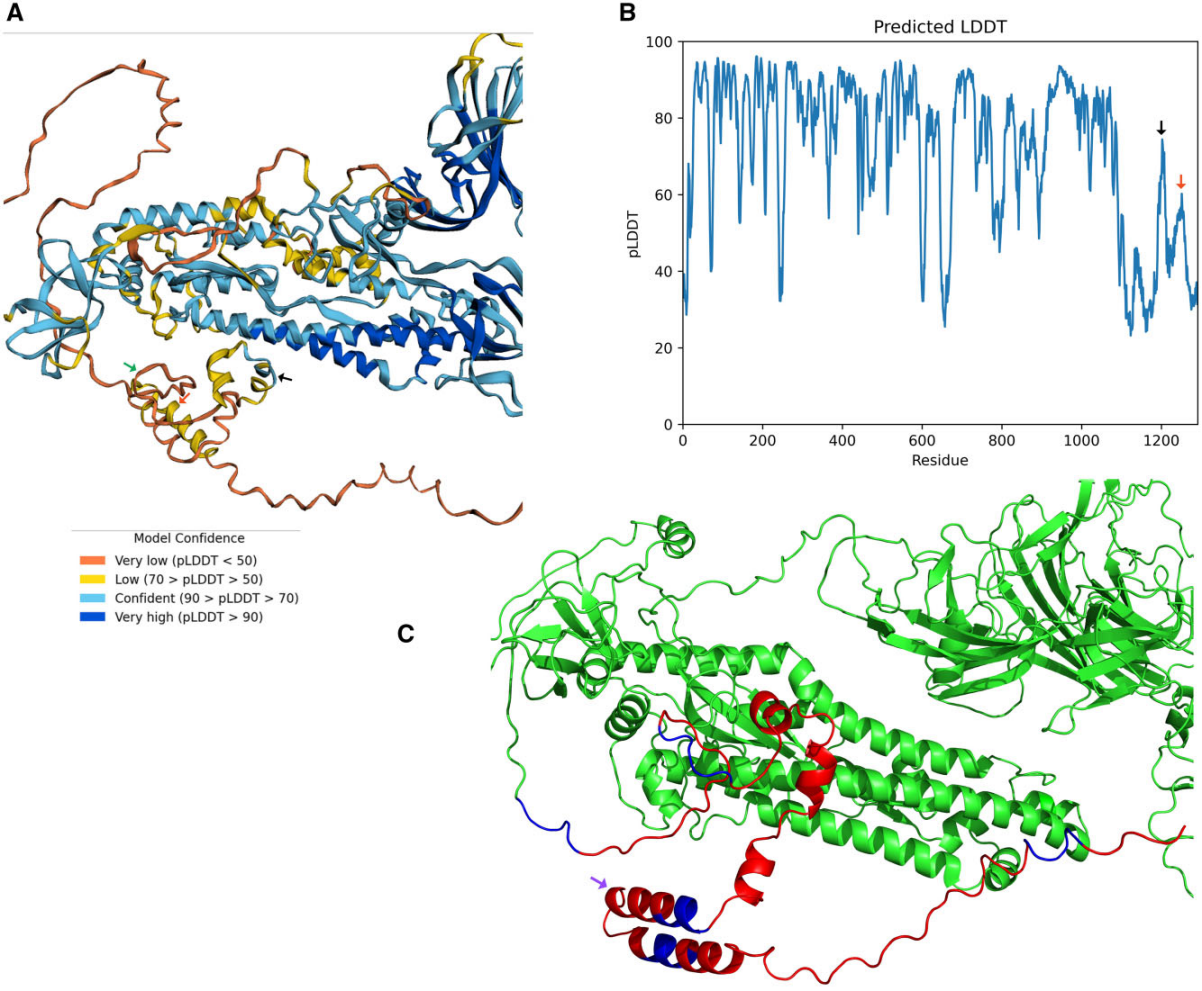
The AlphaFold model of the final vaccine construct (without transmembrane domain). (A) Best model for the Vacdeltm construct and (B) pLDDT plot. Except cytoplasmic domain, most of the other portions of the structures have pLDDT values >70. The model is color coded based on pLDDT confidence scores. In the cytoplasmic domain, the central region of FDE (marked by a black arrow) has pLDDT scores >70 and >50 for terminal portions. Similarly, part of the memory peptide (red arrow marked) was folded with >50 score. As expected from non-complementary β-stranded structure in PDBsum (protein database summaries), the first epitope (DLSPRWYFYYLGTGP) (green arrow marked) forms unstructured region (pLDDT < 50). (C) The envelope peptide between the folding initiator peptide and memory epitope is folded as helices (from PyMOL; Purple arrow marked). The cytoplasmic epitopes and EAAAK linkers are colored red and blue, respectively. The rest of the protein is colored green.

**Figure 9.**
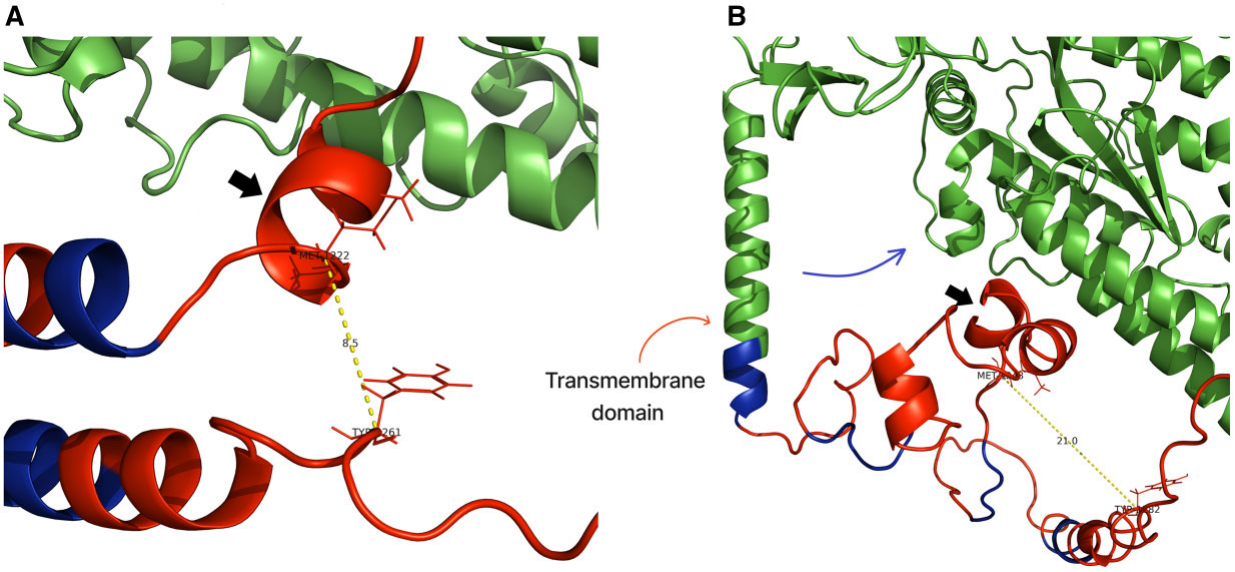
The folding center of the vaccine constructs with T-cell epitopes (red) joined by linkers (blue). (A) Without transmembrane domain (Vacdeltm) and (B) with transmembrane domain (VacFL). The linkers (EAAAK) are colored blue while individual T-epitopes in cytoplasmic domain are colored red. The closest distance between FDE (marked by black arrow) and memory peptide is monitored by the central methionine of the former and a tyrosine residue of the latter. In Vacdeltm, this distance is 8.5 Å which extends to 21 Å in VacFL construct. The transmembrane domain (marked by red arrow) is helical and seems to pull (blue arrow) the adjacent cytoplasmic domain regions (which includes FDE) toward rest of the protein (spike portion, green). The distal region (with memory peptide) of the cytoplasmic domain is not influenced by this pulling effect and stays away from FDE.

**Figure 10.**
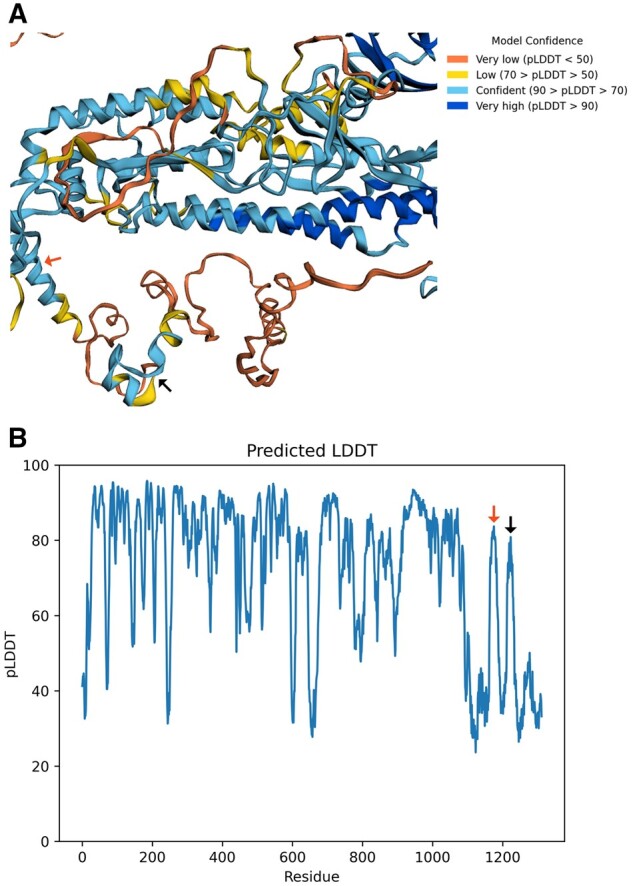
AlphaFold model of the vaccine construct with transmembrane domain (VacFL) and predicted LDDT plot. (A) The best model is shown. The folding initiator epitope is marked by a black arrow. This epitope is well-folded while other epitopes are unstructured in cytoplasmic regions. The transmembrane domain is marked by a red arrow. (B) The pLDDT plot shows that the red arrow marked transmembrane region (with pLDDT > 80) and the other domains are well-folded except the cytoplasmic region. The model confidence for folding driver peptide (denoted by black arrow) is higher (>80) than Vacdeltm indicating no effect from neighboring unstructured epitopes.

**Figure 11.**
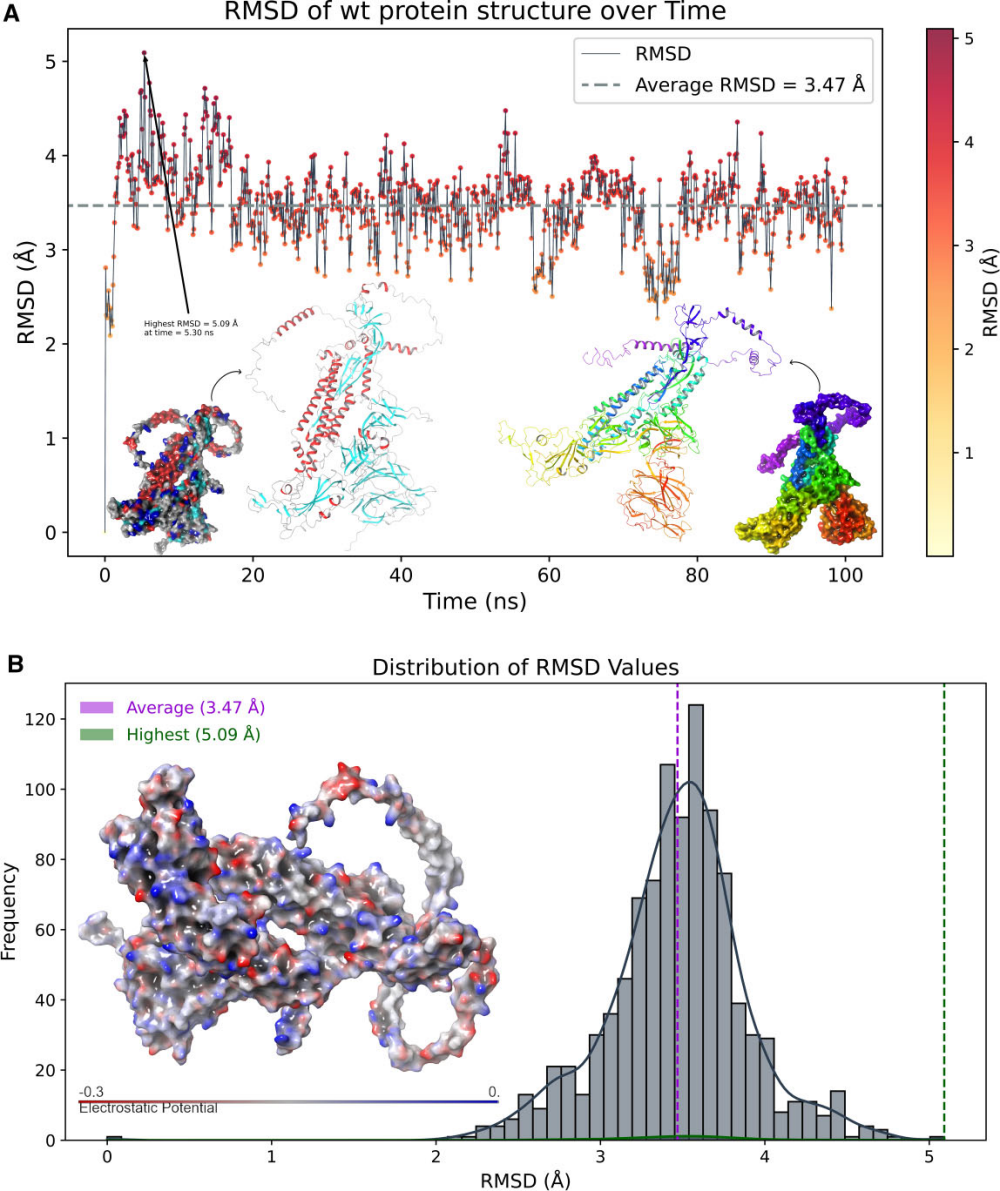
(A) Shows the highest RMSD at 0 ns (5.09 Å) and overall average of 3.47 Å for wild-type spike structure’s Carbon-alpha group atoms. The average line is shown in gray color. (B) Shows the highest RMSD at 0 ns (5.09 Å) and overall average of 3.47 Å for wild-type spike C-alpha group atoms including the electrostatic interactions value ranges within 0 to −0.3 kcal/mol.

**Figure 12.**
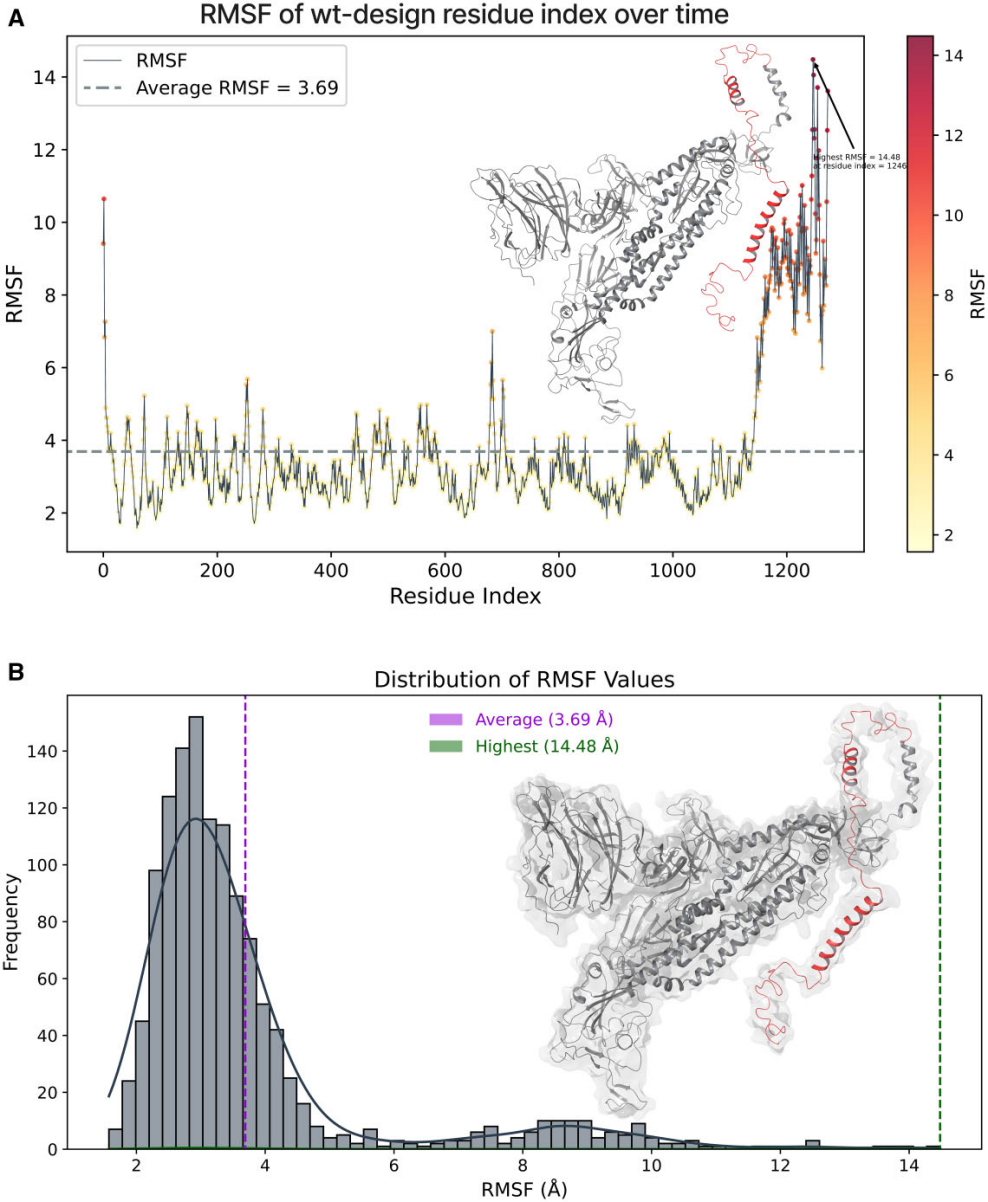
(A) Shows the average 3.69 Å RMSF and highest for single reside at 1246 position 14.48 Å at 100 ns time for wild-type spike. The highly flexible region is represented in red color. (B) Shows the RMSF values in a histogram representation and the average value of 3.69 Å in the pink line color, while the green indicates the highest value of 14.48 Å for wild-type spike at 100 ns trajectory.

**Figure 13.**
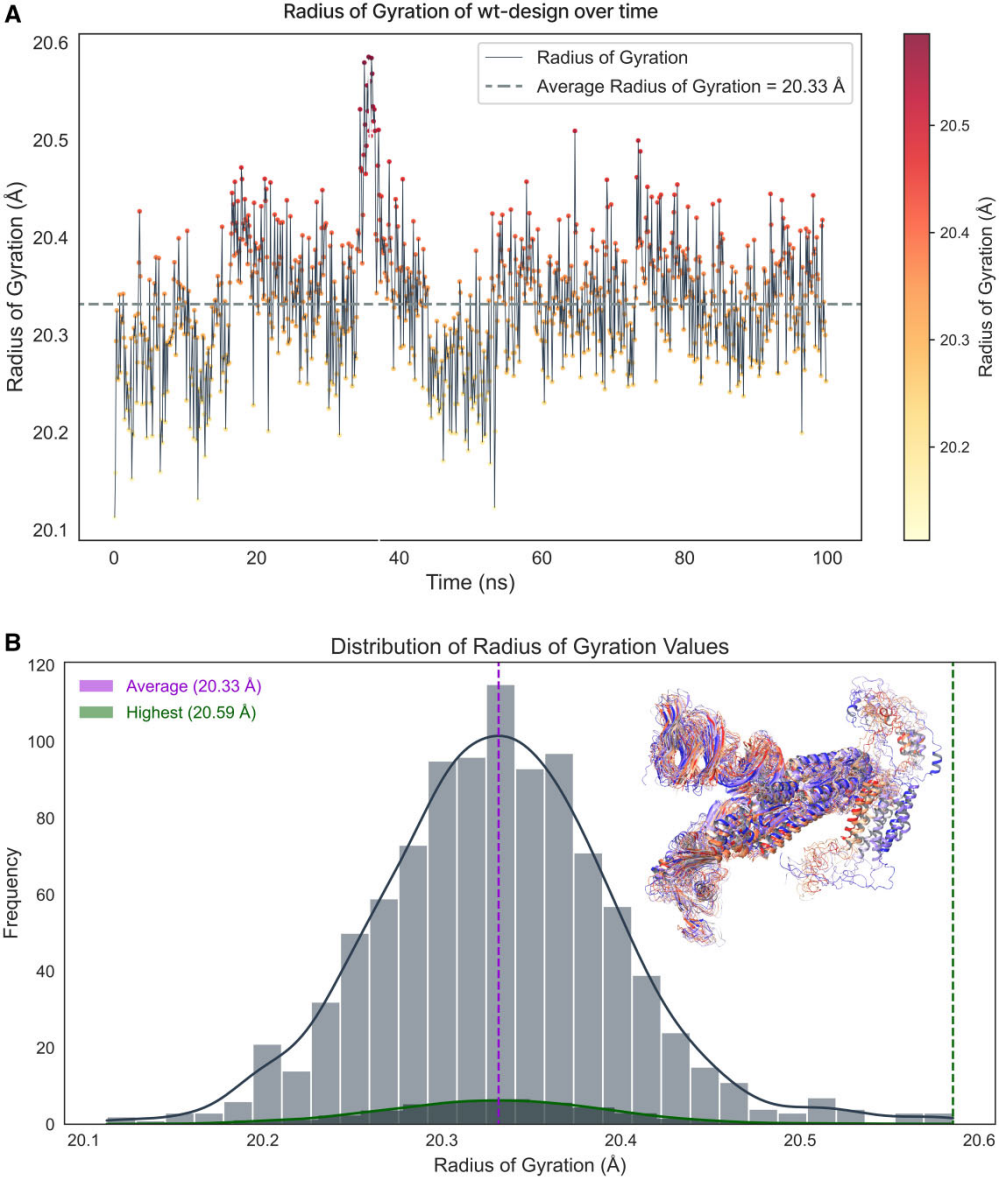
(A) Indicates the Rg for the wild-type of spike at 100 ns time simulations with an average value of 20.33 Å in a gray color line representation. (B) The histogram shows the Rg values of the highest 20.59 Å and average of 20.33 Å in green and pink colors, respectively, for wild-type spikes. Blue to red represents the trajectories from 0 to 100 ns simulation time-based gradients.

**Figure 14.**
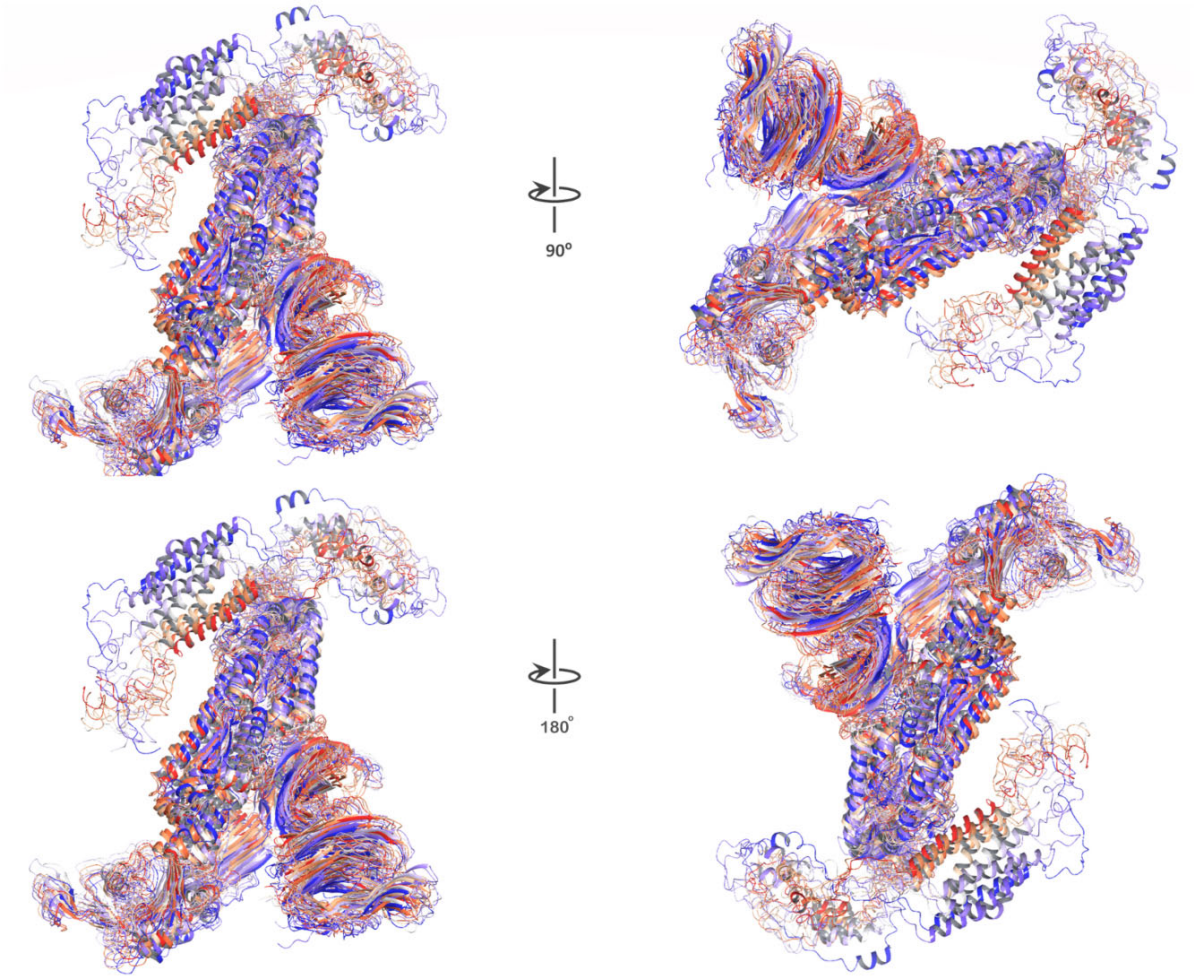
It shows the wild-type of spike structural change over time from 0 ns to 100 ns in two different angles: 90° and 180°. The blue color indicates the initial state, and the red color indicates the final conformation and compactness that the structures achieved after 100 ns of simulation.

## Conclusion

Given the utmost need for potential vaccines against deadly SARS-CoV-2 pathogen candidates recently an emergency left by the World Health Organization (WHO), our study provides comprehensive in-silico strategy to design vaccine against SARS-CoV-2 using the long-lasting potent T-cell immune memory strategy. Our strategy proposes that the Vac^deltm^ construct with linker joined cytoplasmic epitopes can be a potential vaccine construct against the deadly SARS-CoV-2 (VOC-variant of concern), extending its capability further against the future evolving variants. This is further validated in 100 ns simulation revealing the stable overall fold conformation, less fluctuation and transition in terms of structure mobility, and a relatively compact fold conformation throughout the simulation. This strategy is not only applicable for SARS-CoV-2 (VOC) but can also easily be replicated and tweaked for other viruses with the potential to cause pandemics listed by WHO and CDC (Center for Disease Control). This includes human respiratory viruses such as influenza, respiratory syncytial virus, and others with no countermeasures like Ebola, Marburg, Lassa, and Nipah viruses. The non-allergenic CD4^+^ T-cell epitopes from these pathogens can be screened based on similarity to closely related milder viruses against which humans have immunity. The peptides can be strategically placed in linker regions within our designed construct, to create an instant vaccine design. The resulting domain and epitopes’ structural stability can be confirmed through MD and experiments. Therefore, our study provides new insights and a comprehensive in silico protocol for long-lasting potent T-cell immune memory by considering a significant set of mutations and T-cell epitopes from SARS-CoV-2 (VOCs) in the vaccine constructs. However, in-vivo and in-vitro validation (such as live animal testing and cellular assays) of the vaccine construct and other analyses (such as experimental structural determination) are necessary which is lacking in this study. This includes the incorporation of hotspot residues of spike proteins from ever-changing variants to design a pan-coronavirus vaccine. Nonetheless, our study shows a robust computational pipeline for rationally designing a pan-SARS-CoV-2 vaccine construct. With further optimization, this computational approach could accelerate the development of next-generation vaccine candidates with broader and more durable protection against this rapidly mutating virus.

## Future prospects

The epitopes in the vaccine models in our study are from clinical studies. For T-cell CD4^+^ epitope selection in future vaccine designs, structural fold accuracy may be validated through AlphaFold or other structural studies after placing with suitable FDE. We also observed in immunological studies from literature the striking differences in CD4+ epitope immunogenicity when in vitro studies such as (IFN)-γ ELISPOT are conducted. The envelope peptide (a CD4+ epitope) which we have chosen in our design has shown good immunogenicity in one study [36], but negligible immune binding in another study [77]. This anomaly may be due to lack of proper structure which cannot be monitored in the peptide epitopes in solution driven by random movement. During the simulation of Vac^deltm^, we observed selective docking of envelope peptide with spike S2 domain (Supplementary movies; Supplementary Fig. S12B). This epitope anchors with human cellular adhesion protein during infection [64]. Therefore, the intrinsic property of binding to others can be displayed only if it is properly folded which was achieved in Vac^deltm^. For Vac^FL^, neither this epitope was folded nor did it bind to any other region (Supplementary movies). For this construct, cytoplasmic epitopes were more affected by the movement of transmembrane helix and kinetic inertia which positioned the domain laterally to the spike (Supplementary movies). Although the memory epitope was more folded by one more helical turn in this construct than Vac^deltm^. But this folding does not reflect the local strain in this turn which is seen in the latest native nucleocapsid structure (RSCB PDB 8FG2) (Supplementary Fig. S13). So for vaccine design, structural validation of CD4^+^ T-epitopes is important while designing a multiepitope vaccine. Lastly, such vaccine constructs may be placed under antigen-presenting cell (APC)-specific promoter or targeted to APC only to avoid anergy (failure to respond to an antigen, even when antigen-specific cells are present) and undesirable side effects in the long run [78, 79].

## Supplementary Material

bpad021_Supplementary_Data

## Data Availability

The designed vaccines’ structures Vac^deltm^ and Vac^FL^ can be accessed at Protein Model Database PMDB ID: PM0084589 and PMDB ID: PM0084590, respectively. The native spike AlphaFold model is available from the corresponding author if needed. (A and B) Displays the RMSD values of the vaccine construct without transmembrane region (Vacdeltm) structure at 100 ns simulations, the average RMSD is denoted by gray dash line, while the highest is by a black arrow including the histogram representation. In inset, 100 ns structure is shown. The folding interactions created between FDE and other cytoplasmic epitopes are marked by a green arrow. The terminal segment (blue arrow) of the distal memory epitope along with ER export signal remains unstructured as noted in the AlphaFold model. (C and D) Displays the RMSD values of the vaccine construct with trans-membrane region (VacFL) structure at 100 ns simulations, the average RMSD is denoted by gray dash line, while the highest is by a black arrow including the histogram representation. In inset, 100 ns structure is shown. The green arrow indicates the location of the cytoplasmic FDE. Also noted more folding in memory epitope (blue arrow). The terminal portion of the memory peptide along with ER export signal remains unstructured like Vacdeltm. (A and B) Displays the RMSF values of the Vacdeltm structure at 100 ns simulations, the average RMSF is denoted by gray dash line, while the highest is by a black arrow and the histogram representation. (C and D) Displays the RMSF values of the VacFL structure at 100 ns simulations, the average RMSF is denoted by gray dash line, while the highest is by a black arrow and the histogram representation. (A and B) Displays the Rg values of the Vacdeltm structure at 100 ns simulations, the average Rg is denoted by gray dash line, while the highest is by a black arrow and the histogram representation. (C and D) Displays the Rg values of the VacFL structure at 100 ns simulations, the average Rg is denoted by gray dash line, while the highest is by a black arrow and the histogram representation.
