# Supplementary material for: De novo design of anti-variant COVID-19 vaccine
Source: Biol Methods Protoc. 2023 Sep 26;8(1):bpad021. doi: 10.1093/biomethods/bpad021 (PMC10580973; doi:10.1093/biomethods/bpad021)
Supplement: bpad021_Supplementary_Data [file bpad021_supplementary_data.zip › Supplementary_Fig._S_1-13.pdf]

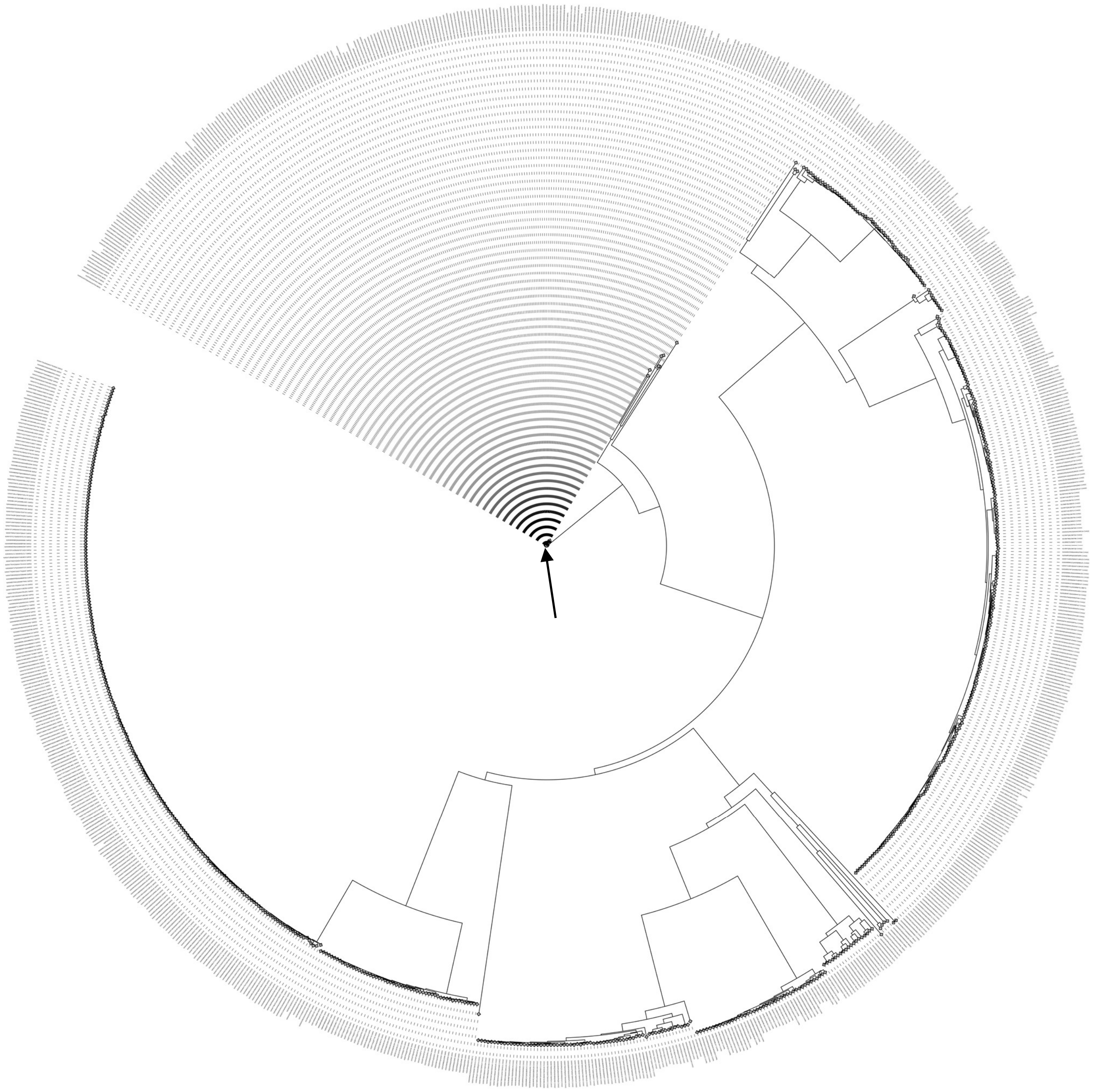

Fig S1: Sequence similarity based neighbouring-joining circular tree of spike proteins from SARS, SARS-2, MERS and common cold coronaviruses. Marked by arrow are MERS spike protein family (chosen from Uniprot after removing fragments, gapped sequences and non-human host infecting MERS coronaviruses) showing very little changes in their sequences as compared to others.

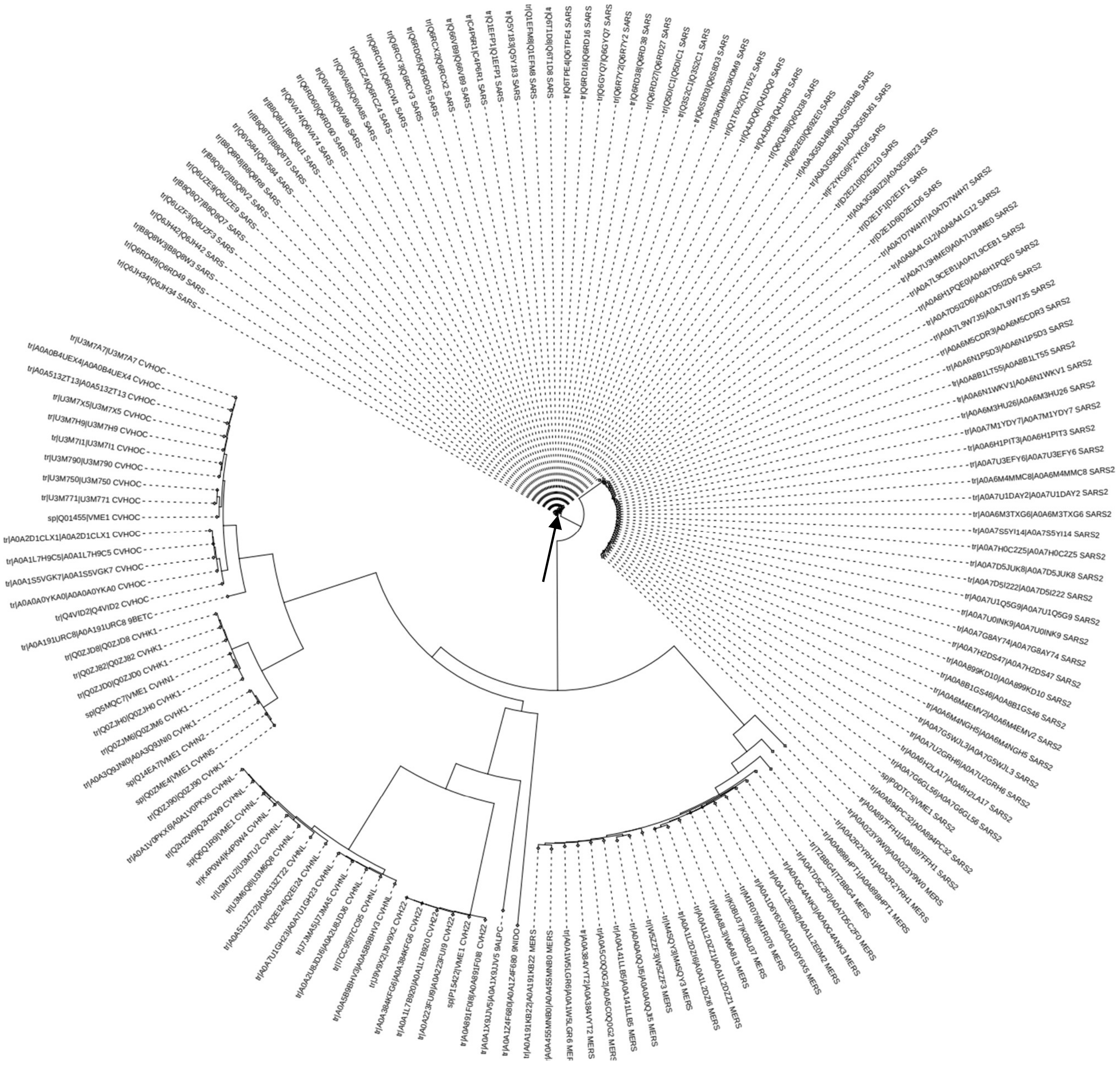

Fig S2: Sequence similarity based neighbouring-joining circular tree of membrane proteins from SARS, SARS-2, MERS and common cold coronaviruses. Indicated by arrow are SARS membrane protein family (chosen from Uniprot after removing fragments, gapped sequences and non-human host infecting coronaviruses) showing very little changes in their sequences as compared to others.

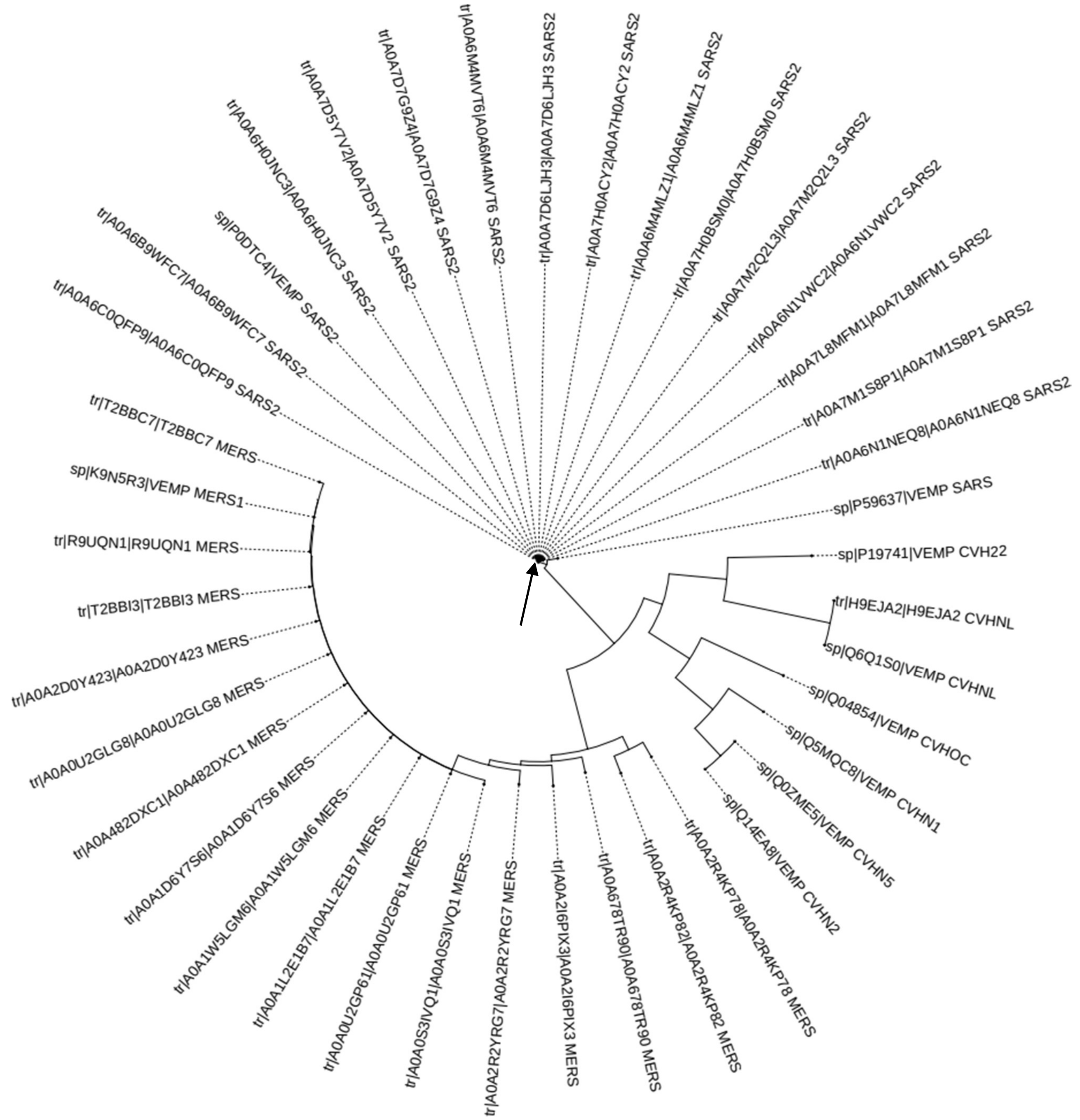

Fig S3: Sequence similarity based neighbouring-joining circular tree of envelope proteins from SARS, SARS-2, MERS and common cold coronaviruses. Marked by arrow are SARS-2 (Covid) envelope protein family (chosen from Uniprot after removing fragments, gapped sequences and non-human host infecting coronaviruses) showing very little changes in their sequences as compared to others. For SARS, only one sequence was found to be without gaps.

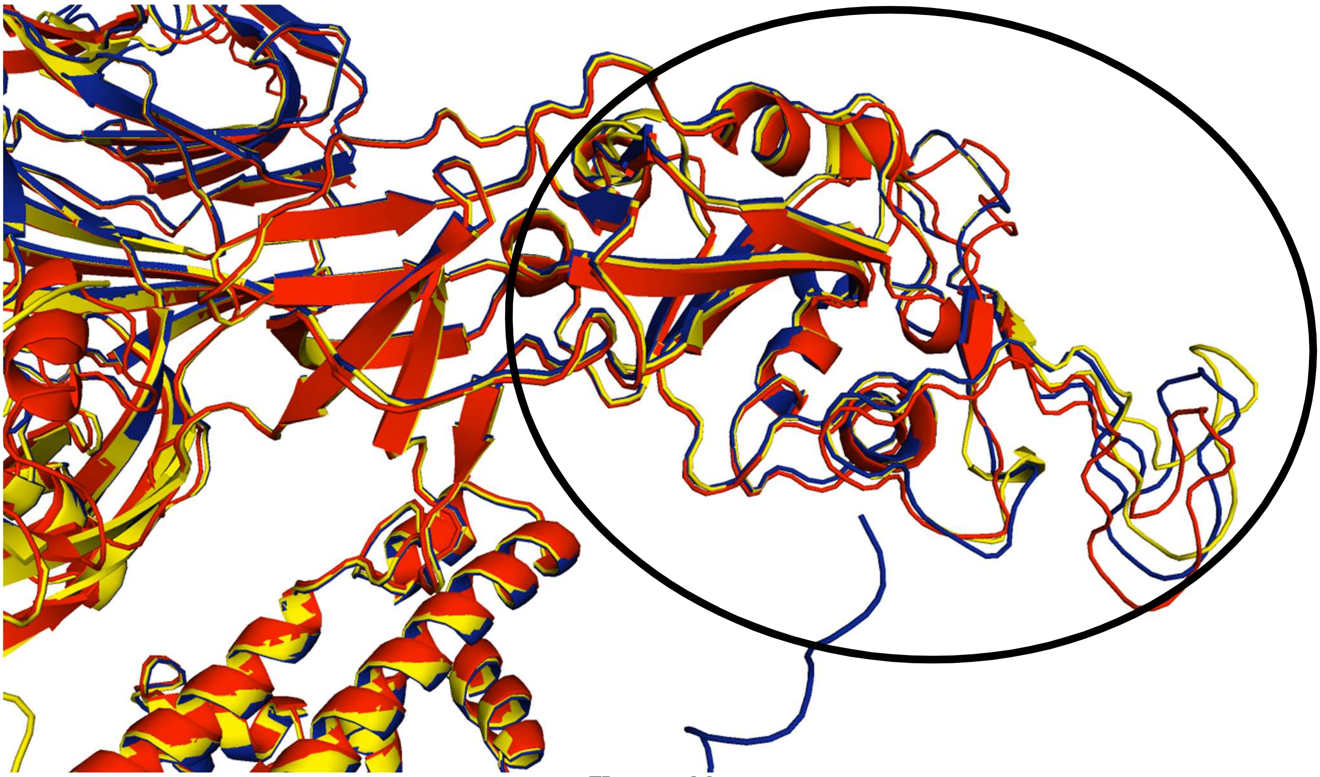

Fig S4: Receptor binding domains of Vac^{FL}, Vac^{deltm} and Native spike models appear nearly identical. Vaccine models with (yellow) and without transmembrane (blue) domain were aligned on native spike model (red) in PyMOL. Encircled are RBDs of all three showing very little changes in their structures.

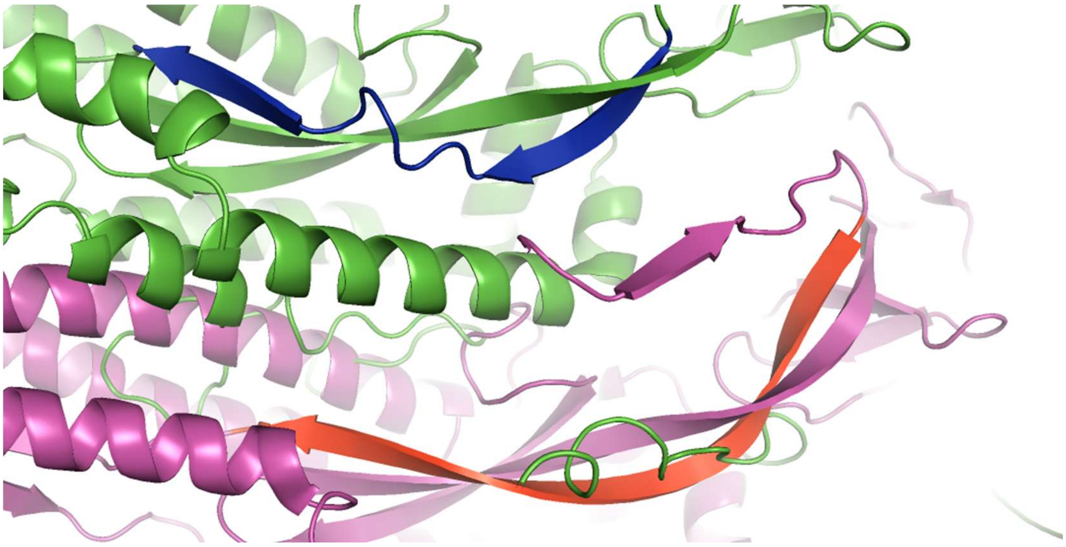

Fig S5: Native core β-strand is replaced by two discontinuous β-strands in vaccine model. The long core β-strand (red) in native spike was deleted in vaccine construct (green) and compared with native spike (magenta). It was observed the lost strand is replaced by two adjacent β-strand stretches

(blue) which can support the core β-sheet in that region. Thus the structural integrity is maintained. But the novel strands' lengths will not be sufficient for interactions with other subunits of trimeric spike to facilitate pre- to post-fusion transition.

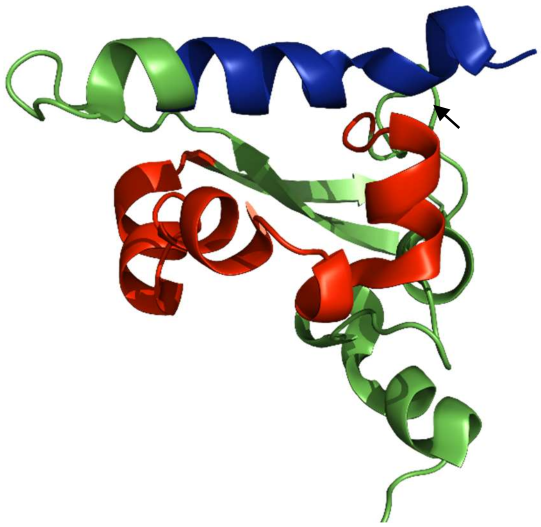

Fig S6: PDB structure of Nucleocapsid protein shows the interaction between epitopes chosen. The pivotal epitope for folding (red) and part of memory epitope (blue) are shown in the structure (6ZCO.pdb). The interacting region is marked by arrow.

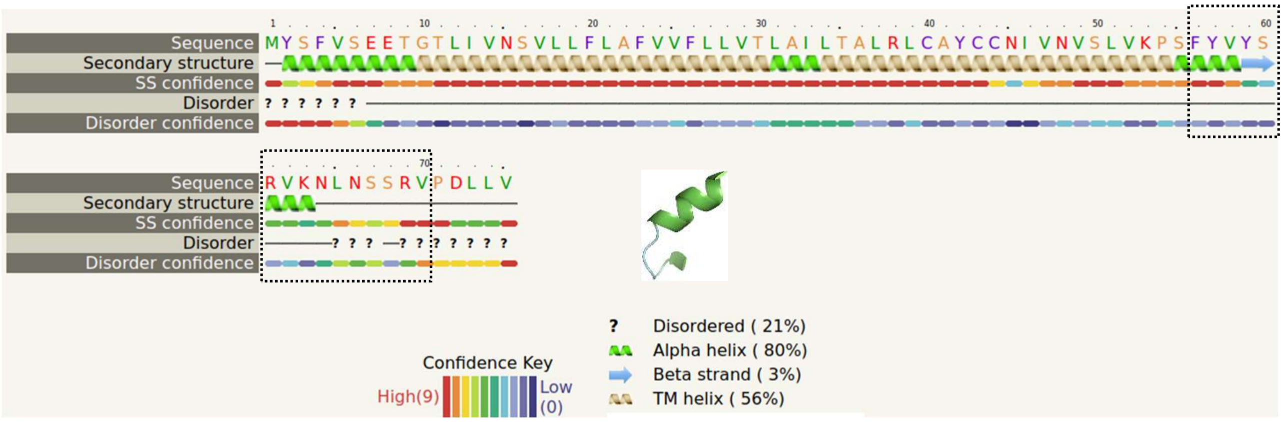

Fig S7: The secondary structure of Envelope protein. Secondary structure motifs of envelope protein are displayed from Phyre2 server. The different secondary structures in whole protein and their percentages are explained in the legend. α-helices are green; β-strands are cyan; transmembrane regions are grey and disordered regions are coloured other than these three shades. Also colour bars represent the confidence values for the different motifs. The chosen epitope peptide in vaccine is marked by dotted box. Two α-helices connected by a short β-strand and a C-terminal disordered region form this peptide. After model building, this epitope was folded with two α-helices interconnected by a short loop in Vac^{deltm} (Inset box on top of legend). There was no folding in case of Vac^{FL}.

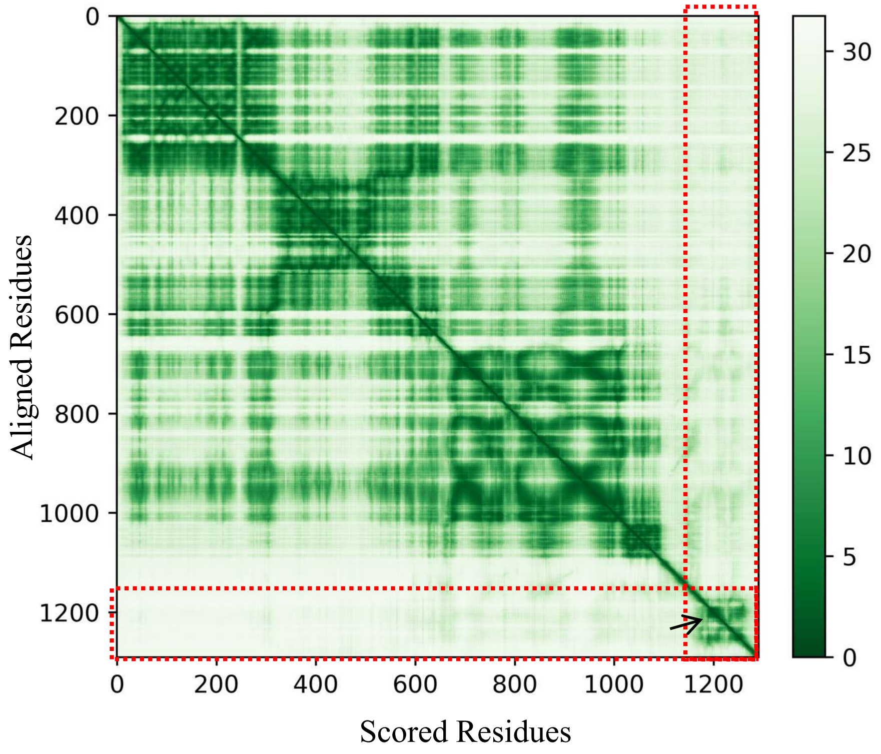

Fig S8: Predicated aligned error (PAE) plot for Vac^{deltm} model. This plot is obtained from monomer pTM (predicated template modelling) method in AlphaFold. Scored residues represent predicted positions of xth residue and aligned residue denote actual positions of yth residue. White gaps indicates maximum positional error (Å). The Accuracy in domain modelling is directly proportional to intensity in green shade in the plot. The colour bar on the right side represents the error levels. Two perpendicular dashed rectangles (red) are drawn on the map which intersects on the diagonal to form a square. Enclosed inside this is the cytoplasmic domain (1165-1291) which is partially disordered**.** Folding driver peptide (1190-1224) is folded with less error (marked by arrow). Adjacent linker and envelope peptide (1230-1244) is also folded with good error values. Towards the end of cytoplasmic domain error increases which indicates unstructured terminus.

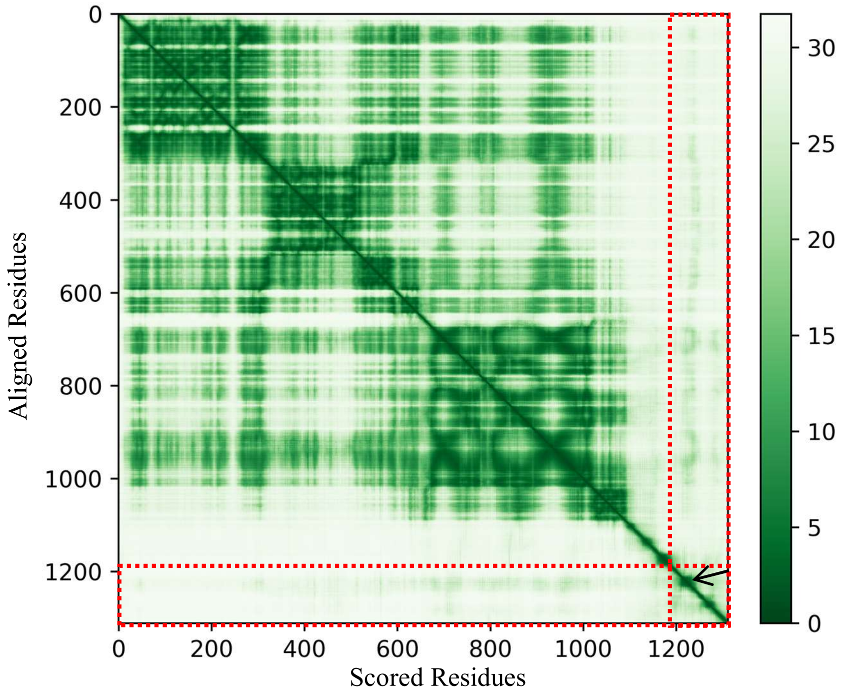

Fig S9: Predicated aligned error (PAE) plot for Vac^{FL} model. Heat map is generated from monomer pTM (predicated template modelling) generated model for Vac^{FL} in AlphaFold. Enclosed inside two criss-crossed dashed rectangles-generated square box (red) is the cytoplasmic domain (1186-1312). The colour bar on the right side represents the error levels. Folding initiator peptide (1211-1245) is folded with less error (intense green colour). Also this epitope is represented by narrow patch (marked by arrow) on the diagonal as compared to broad and diffused area for Vac^{deltm} model in previous figure. This may indicate less effects from neighbouring unstructured regions on this peptide. Overall, in most of the cytoplasmic domain error increases corresponding to disordered structure.

A)

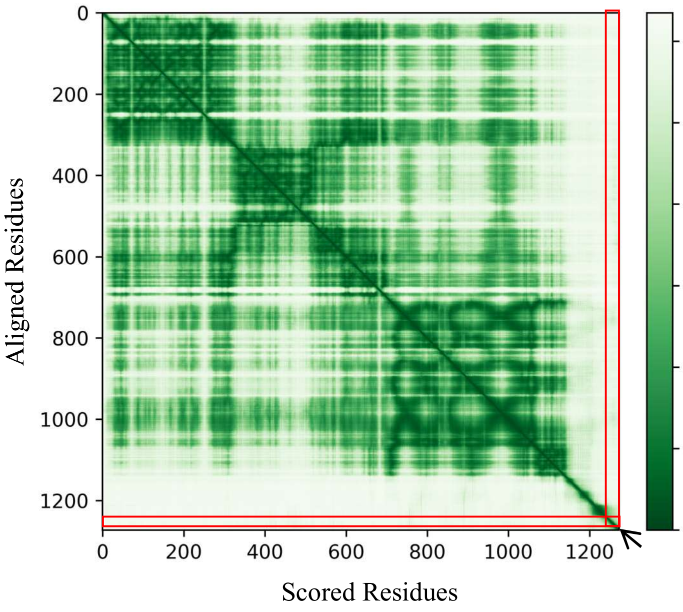

B)

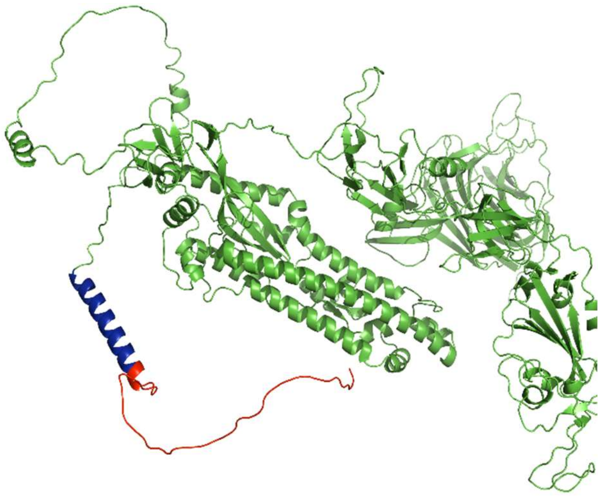

Fig S10: Predicated aligned error (PAE) plot for native spike model. A) The heat map represents relative errors of domain folding for native spike in pTM model building. Enclosed inside the tiny square (denoted by arrow) generated by two intersecting rectangles (red) is the cytoplasmic domain (1235-1273). The colour bar on the right side represents the error levels. In the cytoplasmic domain white shade dominates corresponding to disordered structure. **B)** Native spike model (best model from AlphaFold visualized in PyMOL) with mostly unstructured cytoplasmic domain (red) and α-helical transmembrane domain (blue). Rest of the protein is coloured as green.

A)

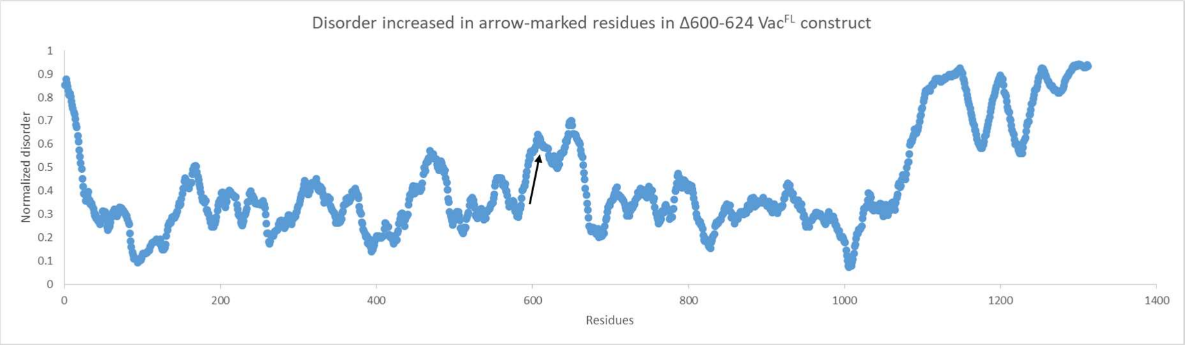

B)

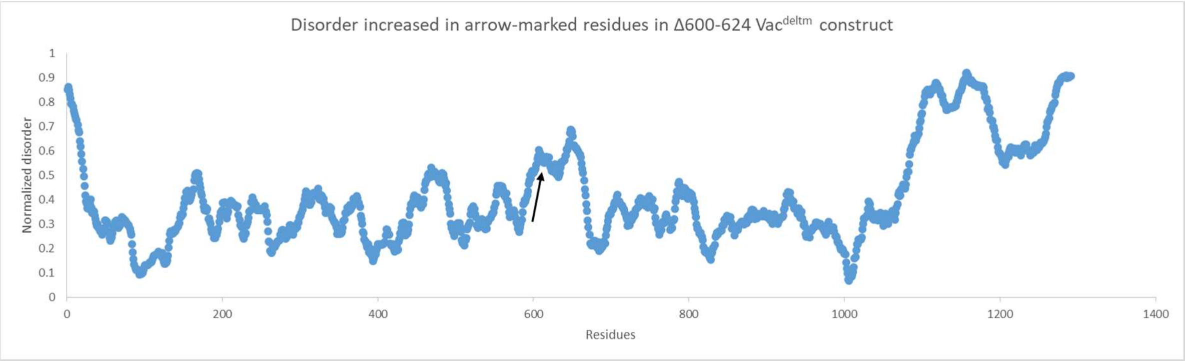

C)

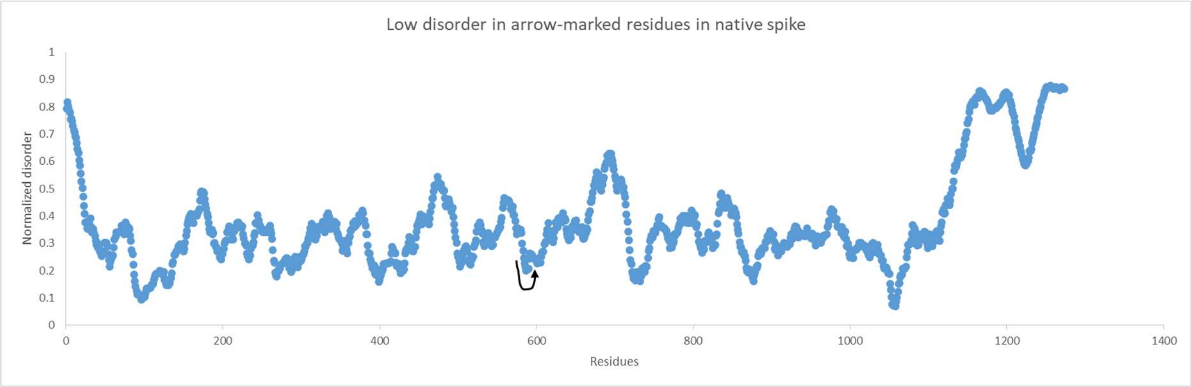

Fig S11: Disorder plots calculated from AlphaFold models indicate increased fluctuations in remaining potential ADE causing region after deletion. Increased disorder shown by arrow in **A)** vaccine construct with transmembrane domain and **B)** vaccine construct without transmembrane

domain while **C)** less disorder is seen in intact ADE region in native spike with transmembrane domain. The disorder per residue were predicted by AlphaFold-disorder package and normalized per 25 residues' frame. The graphs were plotted in MS-Office Excel (2013).

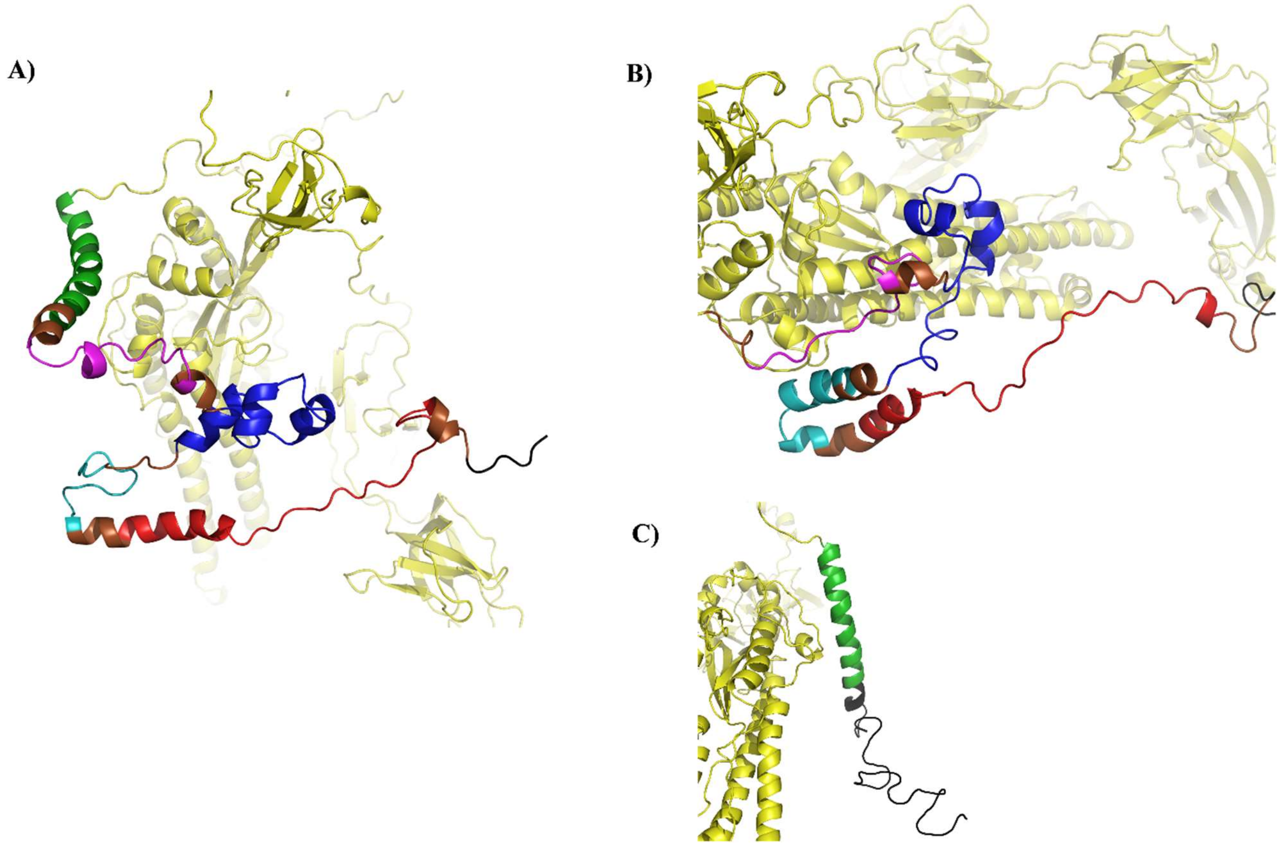

Fig S12: The cytoplasmic domains of AlphaFold models after 100 ns simulation remained stable. The original folds of epitopes are maintained in designed cytoplasmic domains of **A)** Vac^{FL} and **B)** Vac^{deltm}. The color codes in A) and B) are transmembrane domain (green), Linkers (Brown), First unstructured N-epitope (Magenta), FDE (Blue), Envelope epitope (Cyan), Memory epitope (Red) and ER export signal (Black). For Vac^{FL}, slightly more folding is observed in memory peptide than Vac^{deltm}. But the entire envelope epitope remained unstructured in the former. **C)** In native spike, the intrinsic disorder in short cytoplasmic domain (Black) after transmembrane domain (green) is maintained after 100 ns simulation. Other domains are coloured yellow for all structures.

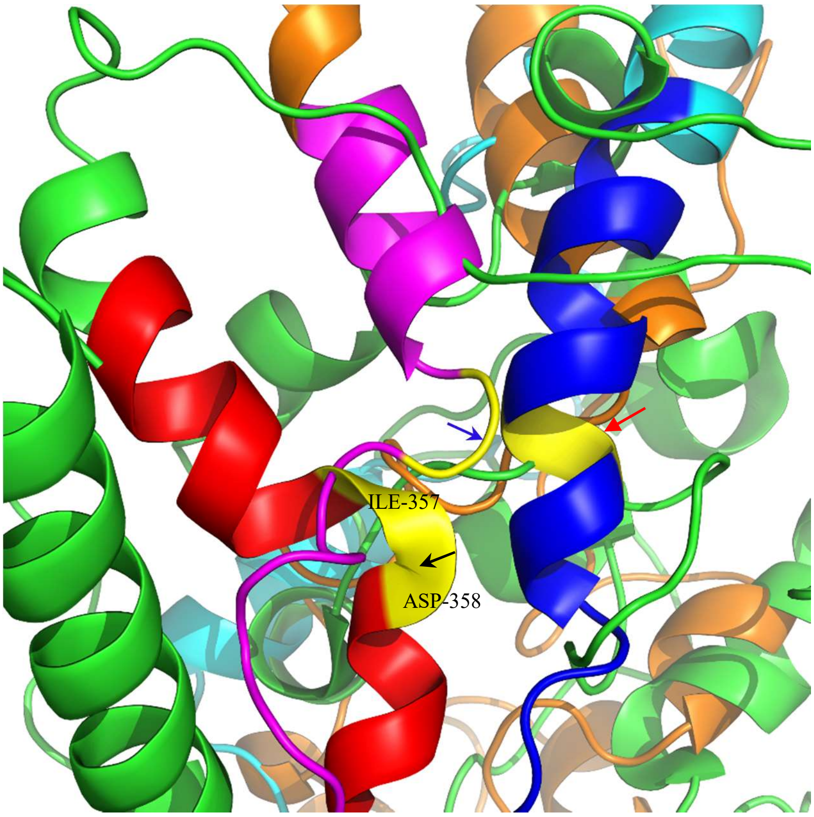

Fig S13: The local strain in native nucleocapsid memory epitope was not visible in post simulation folded segment of Vac^{FL}. The memory epitopes are coloured as red in native nucleocapsid (N) structure (RSCB PDB 8FG2), blue in Vac^{FL} and magenta in Vac^{deltm}. The region affected by neighbouring negatively charged aspartate and hydrophobic isoleucine in memory epitope is coloured as yellow in all. The local strain (denoted by black arrow) in native N- memory peptide helix was not detected in perfectly helical fold (red arrow-marked) of Vac^{FL} memory epitope. The same region (indicated by blue arrow) in Vac^{deltm} did not form helix as expected. Other regions are coloured as green (native N), cyan (Vac^{FL}) and orange (Vac^{deltm}).
