# Supplementary figures and images for: De novo design of anti-variant COVID-19 vaccine
Source: Biol Methods Protoc. 2023 Sep 26;8(1):bpad021. doi: 10.1093/biomethods/bpad021 (PMC10580973; doi:10.1093/biomethods/bpad021)

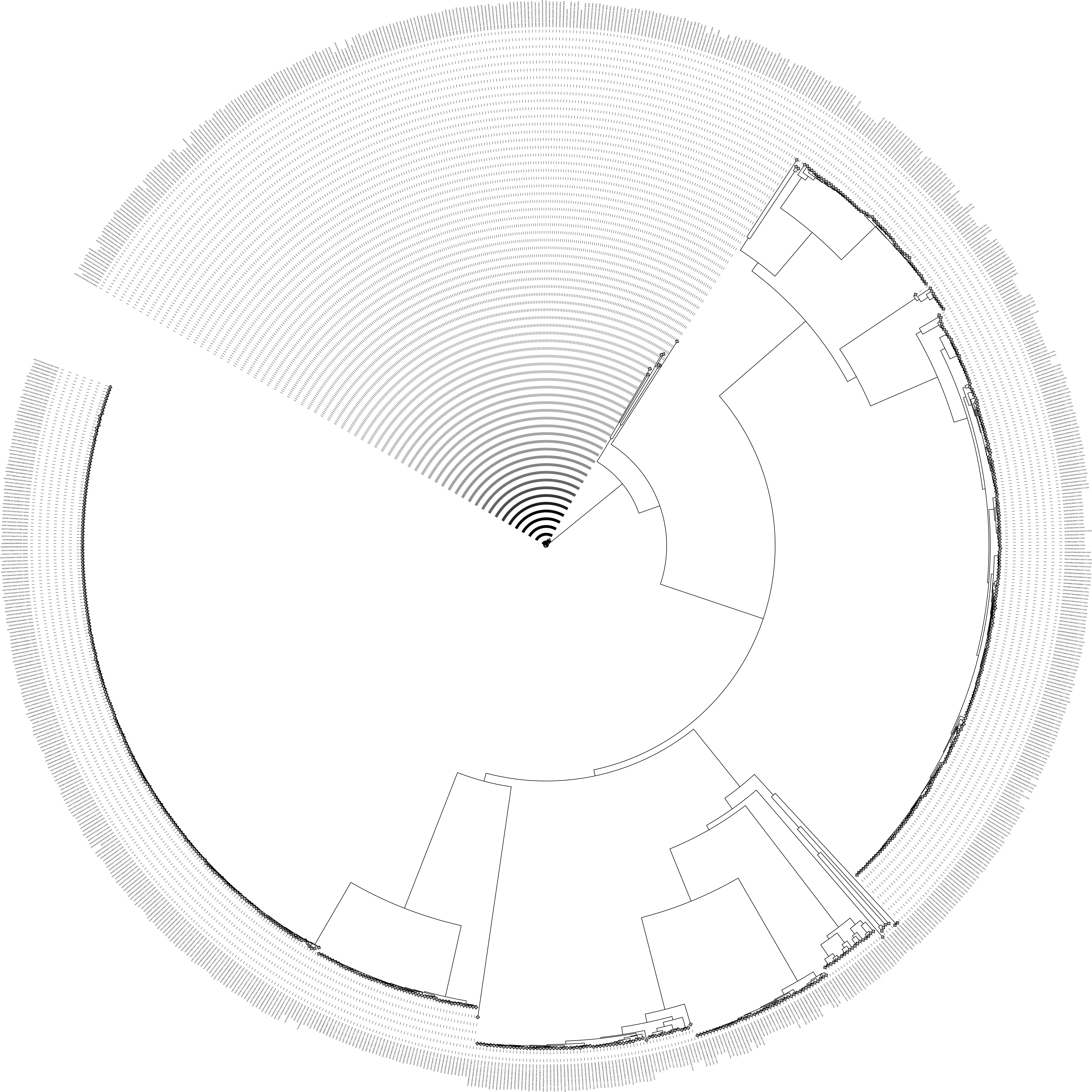

Supplement: bpad021_Supplementary_Data [file bpad021_supplementary_data.zip › Supplementary Fig S1 (Enlarged).pdf]
